# Neuronal activity controls *Bdnf* expression via Polycomb de-repression and CREB/CBP/JMJD3 activation in mature neurons

**DOI:** 10.1038/ncomms11081

**Published:** 2016-03-24

**Authors:** Ernest Palomer, Javier Carretero, Stefano Benvegnù, Carlos G. Dotti, Mauricio G. Martin

**Affiliations:** 1 Departamento de Neurobiología Molecular, Centro Biología Molecular ‘Severo Ochoa' CSIC-UAM, 28049 Madrid, Spain; 2 Laboratorio de Neurobiología, Instituto de Investigaciones Médicas Mercedes y Martín Ferreyra (INIMEC-CONICET-UNC), Universidad Nacional de Córdoba, 5016 Córdoba, Argentina

## Abstract

It has been recently described that in embryonic stem cells, the expression of some important developmentally regulated genes is repressed, but poised for fast activation under the appropriate stimuli. In this work we show that *Bdnf* promoters are repressed by Polycomb Complex 2 in mature hippocampal neurons, and basal expression is guaranteed by the coexistence with activating histone marks. Neuronal stimulation triggered by *N*-methyl-D-aspartate application induces the transcription of these promoters by H3K27Me3 demethylation and H3K27Me3 phosphorylation at Serine 28 leading to displacement of EZH2, the catalytic subunit of Polycomb Repressor Complex 2. Our data show that the fast transient expression of *Bdnf* promoters II and VI after neuronal stimulation is dependent on acetylation of histone H3K27 by CREB-p/CBP. Thus, regulatory mechanisms established during development seem to remain after differentiation controlling genes induced by different stimuli, as would be the case of early memory genes in mature neurons.

## Abstract (web summary)

In neurons, brain-derived neurotrophic factor (BDNF) transcription is activated by synaptic activity, in part by epigenetic regulation of its promoter regions. Here the authors characterize histone modifications in response to NMDA treatment that result in different kinetics of Bdnf activation from its different promoter regions.

## Graphical abstract

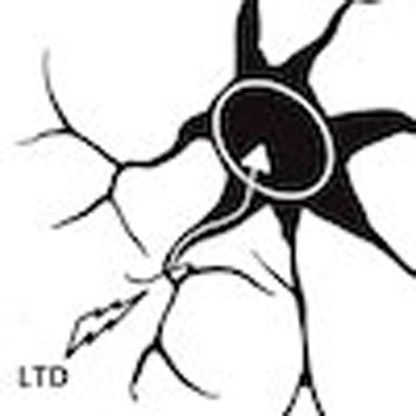

Activity-regulated gene transcription is involved in multiple biological processes, among others, in neuronal survival^1^,^2^ and in the late phases of long-term potentiation (LTP) and long-term depression (LTD), the processes underlying memory formation^3^,^4^. One of the most extensively studied genes that are regulated by neuronal activity is the brain-derived neurotrophic factor (BDNF) which plays important roles in neuronal differentiation, survival and synaptic plasticity^1^,^5^. *Bdnf* transcription is upregulated by membrane depolarization *in vitro*^1^,^6^ and during induction of LTP or associative learning *in vivo*^7^,^8^,^9^,^10^. Neuronal activity is also an important regulator for the temporal and spatial expression of different *Bdnf* isoforms^11^,^12^,^13^,^14^.

The *Bdnf* gene is unusual in that it contains multiple promoters that are specifically regulated under different conditions to generate mRNAs containing different 5′ noncoding exons each of which can be alternatively spliced in a tissue- and stimulus-specific manner to two alternative 3′ exons containing the coding sequence^15^,^16^,^17^. This gene architecture is highly conserved in mammals. In rat and mouse, eight *Bdnf* exons with separate promoters have been characterized^18^,^19^,^20^.

Recent works indicates that synaptic activity regulates *Bdnf* expression through epigenetic control of its promoter regions. For example, contextual fear conditioning in mice results in increased H3 acetylation at the *Bdnf* promoter IV (ref. 21) and defeat stress induces the downregulation of *Bdnf* transcripts IV and VI accompanied by increased H3K27 dimethylation at these promoters^22^. The intimate mechanisms by which different cognition-associated stimuli regulate the activity of specific *Bdnf* promoters are not yet known.

Polycomb repressive complexes (PRC) are required to silence an important subset of developmental regulator genes in both human and mouse embryonic stem (ES) cells, to ensure that expression occurs only at later stages upon ES cell differentiation^23^,^24^. Genome-wide^25^ and candidate-based chromatin studies^26^ indicate that the transcriptional start sites of some of these genes are frequently present in a bivalent state: that is, the presence of histone modifications associated with gene activation (trimethylated H3K4) and with PRC2-mediated gene repression (trimethylated H3K27). A characteristic observed for these promoters in ES cells is that the presence of H3K4Me3 shows a strong positive correlation with the presence of CpG islands in the underlying DNA sequence, whereas the presence of H3K27-trimethylated regions showed also a strikingly low density of transposon-derived sequences (transposon exclusion zones or TEZs)^25^. Interestingly, CpG islands are present at the *Bdnf* promoters of exons I, II, IV and VI and also a transposon exclusion zone was found overlapping these four transcriptional start sites promoters^25^.

We here show that activator and repressor epigenetic marks coexist at *Bdnf* promoters I, II, IV and VI in mature hippocampal neurons and LTD leads to PRC2 derepression of these promoters. The transcriptional profile of these promoters presents two different responses to the stimulus: a slower and stable transcriptional activation of promoters I and IV and the fast transient induction of promoters II and VI. Although the four *Bdnf* promoters share the same regulatory mechanism mediated by polycomb, the fast expression of promoters II and VI also requires H3K27 acetylation. Our data present a novel mechanism involved in the transcriptional regulation of *Bdnf* in mature neurons.

## Results

### Transcriptional analysis of *Bdnf* promoters

**Figure 1:**
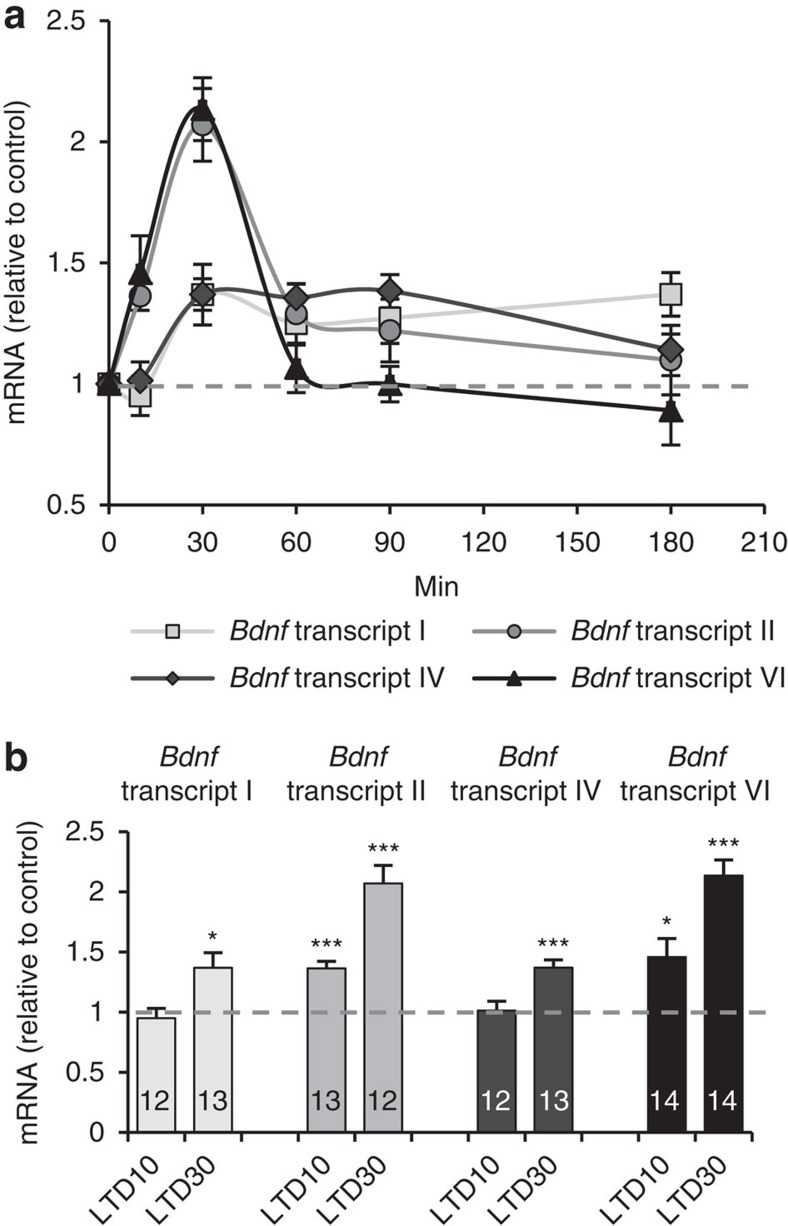
Response of *Bdnf* promoters to NMDA stimulation in mature hippocampal neurons. (**a**) Real-time qPCR analysis of the levels of *Bdnf* mRNAs transcribed from promoters I, II, IV and VI after NMDA stimulation (8–14 independent experiments). (**b**) The bar plot shows the relative amount of each transcript compared with non-stimulated controls at 10 or 30 min after NMDA addition. Data are represented as mean±s.e.m. Statistical analysis by Kruskal–Wallis test and subsequent multiples comparisons by Mann–Whitney U-test with Bonferroni adjustment (*n*: independent experiments or biological replicates; *P<0.05; **P<0.01, ***P<0.001; for statistical analysis see Supplementary Table 1).

We first determined if low doses of *N*-methyl-D-aspartate (NMDA), a stimulus known to lead to LTD^27^,^28^,^29^,^30^, is able to induce expression changes of *Bdnf* promoters. Hippocampal neurons maintained in culture for 2 weeks were exposed during 5 min to 20 μM NMDA and the expression levels of exons I, II, IV and VI were determined by quantitative PCR (qPCR) at different time-points after stimulation. Figure 1a,b shows that a significant increase was observed for transcripts II and VI at 10 min after NMDA addition. The amount of these mRNAs reached a peak at 30 min after stimulation and returned to basal levels at later time-points: 60 min for mRNA VI and 180 min for mRNA II. The expression of exons I and IV, however, presented a different behaviour since a significant increase of these mRNAs was observed only after 30 min upon stimulation. The levels of mRNA I remained at maximum along the entire length of time analysed (180 min), while the levels of mRNA IV returned to basal at 180 min after NMDA application.

These results reveal that chemically induced LTD (NMDA-LTD) triggers two different responses of these *Bdnf* promoters, a fast response of promoters II and VI driving a transient expression of *Bdnf* exons II and VI and a slower response of promoters I and IV, leading to a more stable expression of exons I and IV.

### Epigenetic marks found at *Bdnf* promoters in mature neurons

**Figure 2:**
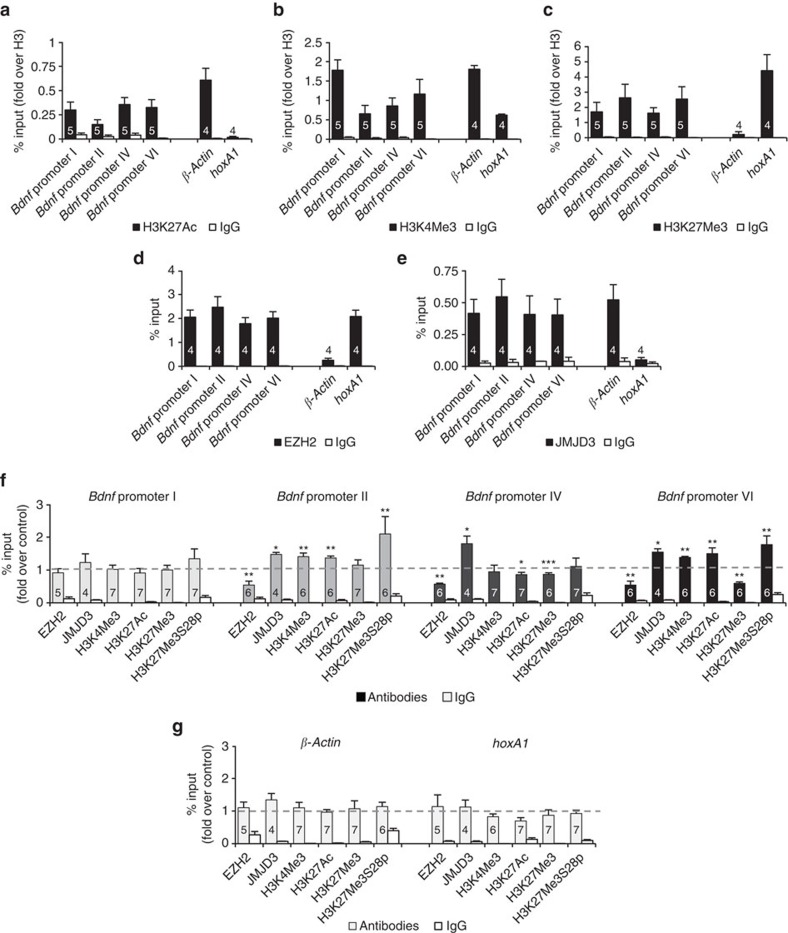
NMDA stimulation induces epigenetic remodelling of *Bdnf* promoters. (**a**–**e**) ChIP-qPCR analysis of epigenetic marks and regulatory proteins bound at *Bdnf* promoters in basal conditions: (**a**) H3K27Ac, (**b**) H3K4Me3, (**c**) H3K27Me3, (**d**) EZH2 and (**e**) JMJD3. The plots also show the levels of the respective marks found at the promoter region of two control genes: a constitutively transcribed *βActin* and a repressed gene *hoxA1*. (**f**) ChIP-qPCR analysis showing the changes in the levels of these proteins at the *Bdnf* promoters measured 10 min after NMDA stimulation. The phosphorylation of H3K27Me3 at Serine 28, which is reported to displace the repressor EZH2, is also analysed. (**g**) ChIP-qPCR analysis shows that the levels of these proteins do not change 10 min after NMDA stimulation at control *βActin* and *hoxA1* promoters. Data are represented as mean±s.e.m. Statistical analysis by Mann–Whitney U-test (the value inside the bars corresponds to *n*, number of cultures used in independent experiments; *P<0.05; **P<0.01, ***P<0.001; for statistical analysis see Supplementary Table 2).

As commented in the Introduction, the structure and genetic environment of *Bdnf* promoters I, II, IV and VI suggest that they are potential sites to be controlled by repressive (PRC) and activating (H3K4Me3) marks. Hence, we next assessed if activator trimethyl-H3K4 (H3K4Me3) and repressor trimethyl-H3K27 (H3K27Me3) are present at *Bdnf* promoters under non-stimulated conditions. In addition, we also searched for the presence of acetyl-H3K27 (H3K27Ac) as an activation form of the H3K27 mark. Chromatin immunoprecipitation (ChIP) assays followed by promoter-specific qPCR performed in non-stimulated hippocampal neurons revealed that these three histone marks are present in all the four *Bdnf* promoters (Fig. 2a–c).

The amount of each histone mark at *Bdnf* promoters was compared with the amount bound to the promoters of two control genes, *βActin* and *hoxA1*. In fact, the constitutively active β-*Actin* presents high levels of activator marks H3K27Ac and H3K4Me3 (Fig. 2a,b) and low levels of the repressive H3K27Me3 mark (Fig. 2c). In contraposition, the constitutively repressed *hoxA1* showed high levels of the repressive H3K27Me3, low amounts of H3K4Me3 and undetectable H3K27Ac (Fig. 2a–c).

To further substantiate that *Bdnf* promoters I, II, IV and VI are repressed by PRC in non-stimulated neurons, we determined the level of association to Enhancer of zeste homologue 2 (EZH2), the catalytic subunit of the PRC2, to these promoters. ChIP experiments revealed the presence of EZH2 associated to the four *Bdnf* promoters (Fig. 2d) at levels that are comparable to the levels bound to the PRC2 repressed gene *hoxA1*. Strikingly, a similar ChIP experiment revealed that, different from *hoxA1*, high levels of the H3K27Me3-demethylase Jumonji domain containing-3 (JMJD3) were present at these *Bdnf* promoters suggesting that these promoters are ready to be de-repressed by H3K27Me3 demethylation (Fig. 2e).

### NMDA leads to epigenetic remodelling at *Bdnf* promoters

To test if NMDA-LTD induces quantitative changes in the different histone marks at our target promoters, similar ChIP experiments were performed in 2-week-old hippocampal neurons in culture 10 min after NMDA stimulation. Figure 2f shows the result of this study: the activation marks H3K4Me3 and H3K27Ac, as well as the JMJD3 demethylase, all increased by the LTD stimulus at promoters II and VI. Furthermore, NMDA treatment resulted in lower levels of the repressor EZH2 at promoters II and VI and reduction of the repressive H3K27Me3 mark at promoter VI. These results may explain the enhanced expression of these transcripts after NMDA stimulation. On the other hand, no change in the levels of these marks were detected at promoter I after stimulation, according to the later expression time of this transcript (30 min, see Fig. 1a). However, in promoter IV, which also undergoes a delayed increased expression (see Fig. 1a), significant decreased levels of repressor EZH2 were accompanied by recruitment of the H3K27Me3-demethylase JMJD3 and decreased amount of H3K27Me3, which would imply de-repression at this early time. However, different from promoters II and VI, no increase of the activator marks H3K4Me3 and H3K27Ac was observed at this promoter implying that both, increase of activation marks and decreased repressive marks, are required for fast transcriptional induction.

Changes were not observed in the levels of these epigenetic marks at the promoter regions of the control genes *βActin* and *hoxA1*, which are not regulated by NMDA (Fig. 2g).

ChIP experiments performed 30 min after NMDA stimulation showed that the levels of all histone marks and their upstream regulators were restored to basal conditions. Significantly, lower levels of H3K27Ac, compared with non-stimulated controls, were observed at promoters II, IV and VI (Supplementary Fig. 1A), suggesting that H3K27Ac deacetylation is involved in the silencing of these promoters after stimulus. However, the possibility of a general effect triggered after NMDA-LTD could not be discarded since reduced levels of H3K27Ac were also observed at the control genes 30 min after NMDA application (Supplementary Fig. 1B).

### Derepression of *Bdnf* requires H3K27Me3S28 phosphorylation

It has been reported that p38 MAPK and mainly its downstream kinases, mitogen- and stress-activated kinases (Msk1 and Msk2), lead to gene activation through H3K27Me3 phosphorylation at Serine 28, the consequent displacement of the SUZ12 subunit of PRC2 and promoter derepression^31^. That was proposed as a mechanism required to activate a subset of Polycomb group of proteins (PcG) target genes by stress-signalling in diploid human fibroblasts and after differentiation signals in human embryonic teratocarcinoma cells^31^. ChIP experiments revealed that H3K27Me3S28 phosphorylation occurs at *Bdnf* promoters II and VI after 10 min of NMDA stimulation (Fig. 2f), suggesting that a similar mechanism may participate during NMDA-induced *Bdnf* expression in mature neurons. In the Supplementary Fig. 2A we show that the antibody used in our experiments presents a weak cross-reaction with H3K9Me3S10p, much less than previously reported for other rabbit polyclonal antibodies for H3K27Me3S28p (refs 31, 32).

**Figure 3:**
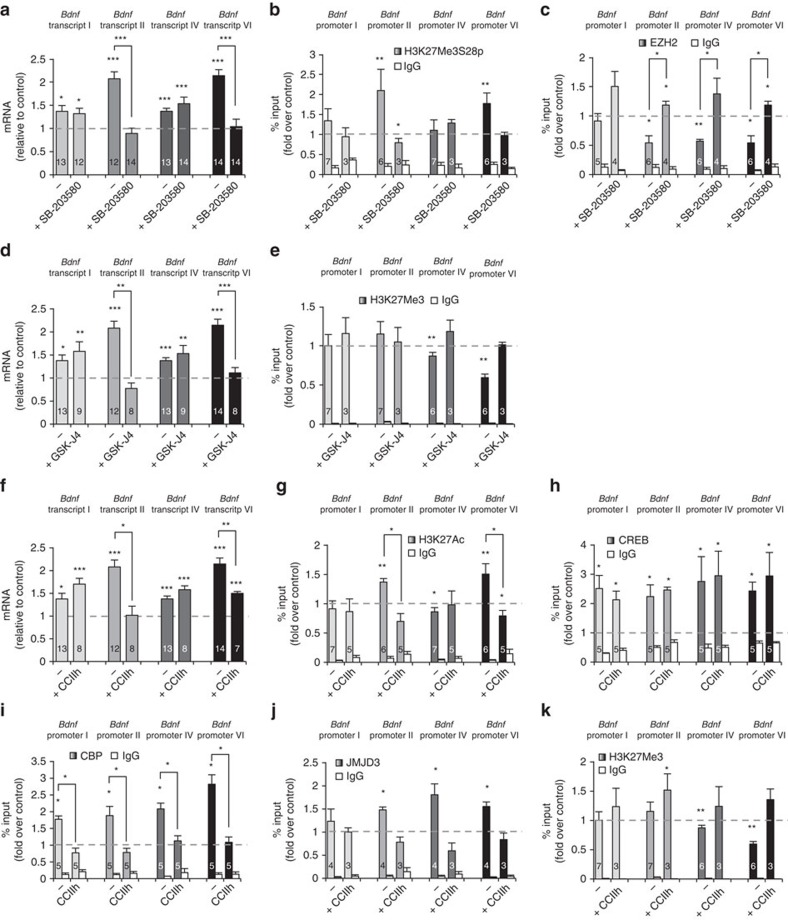
LTD-induced transcription of *Bdnf* promoters II and VI requires EZH2 displacement and H3K27Me3 demethylation and acetylation. (**a**) Real-time qPCR analysis of the *Bdnf* transcripts shows that treatment with the p38 inhibitor SB-203580 blocks the increase of mRNAs transcribed from promoters II and VI at 30 min after NMDA stimulation. (**b**,**c**) ChIP-qPCR analysis performed 10 min after stimulation shows that SB-203580 impairs H3K27Me3S28 phosphorylation at promoters II and VI (**b**). Interestingly, SB-203580 blocked EZH2 displacement from the four promoters studied suggesting a role for EZH2 in the transcriptional control of these regions (**c**). (**d**) The real-time qPCR analysis show that the H3K27Me3-demethylases inhibitor GSK-J4 impairs the NMDA-triggered increase of *Bdnf* mRNAs II and VI observed 30 min after stimulation. (**e**) ChIP-qPCR studies confirm that H3K27Me3 demethylation observed at *Bdnf* promoters IV and VI is blocked by GSK-J4. (**f**) Real-time qPCR showing that the increased expression of *Bdnf* mRNAs II and VI observed 30 min after NMDA stimulation is impaired when CREB-CBP interaction is blocked by CCIIh. (**g**–**k**) ChIP-qPCR analysis show that treatment with CCIIh: blocked H3K27 acetylation at promoters II and VI (**g**), it does not block CREB binding to *Bdnf* promoters (**h**), it prevents CBP recruitment to promoters I, II, IV and VI (**i**) it prevents JMJD3 recruitment at promoters II, IV and VI (**j**) and H3K27Me3 demethylation at promoters IV and VI (**k**). Data are represented as mean±s.e.m. Statistical analysis by Kruskal–Wallis test and subsequent multiples comparisons by Mann–Whitney U-test with Bonferroni adjustment (the value inside the bars corresponds to *n*, number of cultures used in independent experiments; *P<0.05; **P<0.01, ***P<0.001; for statistical analysis see Supplementary Table 3).

To assess if the p38 MAPK pathway may be involved in H3K27Me3S28 phosphorylation in mature neurons, we treated NMDA-stimulated hippocampal neurons with the p38-specific inhibitor SB-203580 (refs 33, 34, 35).The qPCR analysis revealed that this treatment prevented NMDA-induced increase of transcripts II and VI (Fig. 3a). Moreover, ChIP experiments showed that p38 MAPK inhibition abolished NMDA-induced H3K27Me3S28 phosphorylation at promoters II and VI (Fig. 3b). According to these results, ChIP assays performed using a ChIP-validated antibody against p38 (Supplementary Fig. 2B) showed that LTD-induced recruitment of p38 to promoters II and VI at 10 min after stimulation (Supplementary Fig. 2C,D).

In further support that phosphorylation of H3K27Me3S28 is required for Polycomb removal, p38 inhibition precluded EZH2 displacement from *Bdnf* promoters II and VI after NMDA stimulation (Fig. 3c). The expression of exons I and IV was not affected by the treatment with the inhibitor (Fig. 3a,b), further strengthening our early assumption that promoters I and IV are controlled by different mechanisms. Although the SB-203580 also blocked the release of EZH2 from promoters I and IV (change not significant for promoter I), increases in H3K27Me3S28p were not observed in these two promoters after NMDA stimulation, further strengthening the notion that both de-repression and activation are required for NMDA-induced *Bdnf* expression.

Altogether, these data show that the p38 pathway is involved in H3K27Me3S28 phosphorylation and required for the displacement of EZH2 from promoters II and VI after NMDA treatment. Gehani *et al*.^31^ have demonstrated that Msk1/2 kinases are required for H3K27Me3S28 phosphorylation in human fibroblasts. Furthermore, by *in vitro* experiments these authors have shown that Msk2 kinase phosphorylates H3K27Me3S28 more efficiently than p38. Hence, it appears reasonable to conclude that p38 could lead to H3K27Me3S28 phosphorylation by a dual, direct and indirect (via its downstream kinases Msk2 and Msk1) mechanism. Moreover, the results presented here also suggest that the EZH2 displacement could be involved, but is not sufficient, to induce the de-repression of promoters I and IV.

### JMJD3 recruitment to *Bdnf* is required for activation by LTD

Our ChIP experiments showed that JMJD3 demethylase is recruited to promoters II, IV and VI after LTD induction (Fig. 2f). To test if JMJD3 demethylase activity is in fact playing a role for the transcriptional induction of these *Bdnf* promoters, ChIP and qPCR were performed in hippocampal neurons treated with the Jumonji H3K27Me3-demethylase inhibitor GSK-J4 (ref. 36) at the time of stimulation. The qPCR experiment revealed that GSK-J4 inhibited the transcriptional activation of promoters II and VI but had no effect on promoters I and IV (Fig. 3d). The ChIP experiments confirmed that GSK-J4 treatment impaired the decrease of H3K27Me3 observed after NMDA on promoters IV and VI (Fig. 3e). The absence of changes in the levels of H3K27Me3 at promoter II at 10 min after stimulation suggests either that H3K27Me3 demethylation at this promoter occurs at a lower extent than at promoter VI or that it is not detected at 10 min after stimulation. Again, the increase of mRNAs containing exons I and IV was unaffected by the treatment, further supporting the notion that these promoters are controlled by a different regulatory mechanisms. As a matter of fact, even if the GSK-J4 prevented H3K27Me3 demethylation at promoter IV (Fig. 3e), it did not block its activation (Fig. 3d).

The GSK-J4 inhibitor is able to block the catalytic activity of the KDM6 demethylase proteins but also blocks the activity of demethylases from the KDM2, KDM3, KDM4 and KDM5 subfamilies^37^. KDM3 and KDM4 proteins demethylate the repressor mark H3K9Me3, meaning that inhibition of these proteins by GSK-J4 could block induction of the *Bdnf* promoters after LTD; however, by ChIP experiments we found that no H3K9Me3 demethylation is observed at any of the four *Bdnf* promoters at 10 min after NMDA application (Supplementary Fig. 3A).

In agreement with the pharmacological results, the qPCR analysis show that JMJD3 knockdown blocked the NMDA-mediated *Bdnf* induction of transcripts II and VI (Supplementary Fig. 3B–E). H3K27Me3 demetylation was also blocked by JMJD3 knockdown (Supplementary Fig. 3F). All together, these results confirm that JMJD3 is involved in H3K27Me3 demethylation after NMDA-LTD.

**Figure 4:**
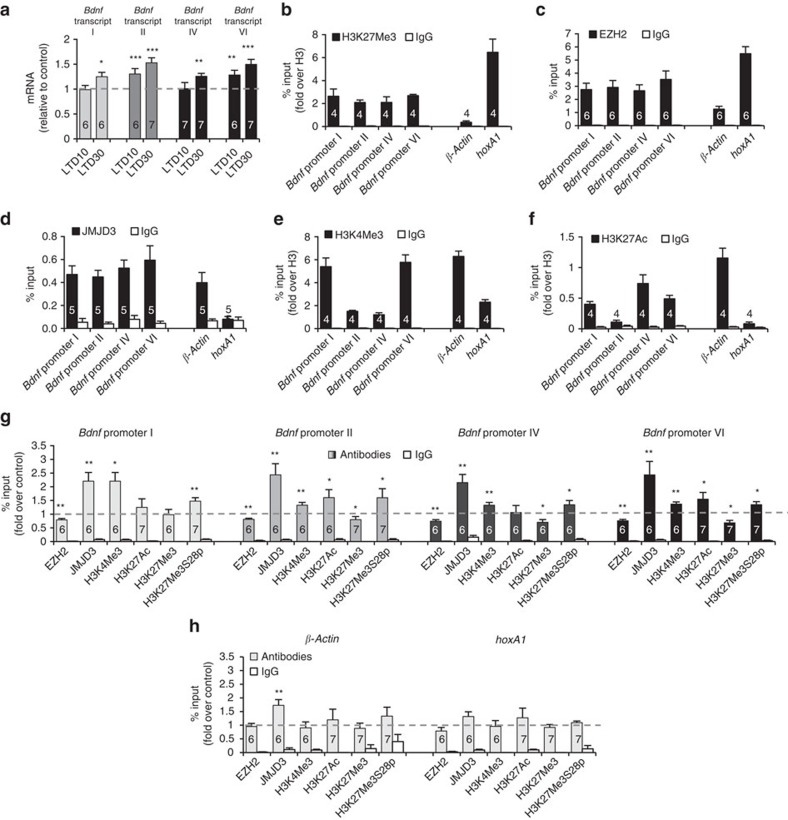
Experiments performed in acute hippocampal slices validate the results *in vitro*. (**a**) According to the data *in vitro*, real-time qPCR analysis performed in acute hippocampal slices from adult mice show that NMDA stimulation triggers the increase of the *Bdnf* mRNAs transcribed from promoters I, II, IV and VI at 30 min after stimulation. The data represent the mRNA levels relative to unstimulated controls. (**b**–**f**) ChIP-qPCR analysis performed in acute hippocampal slices from adult mice. The presence of activator and repressor histone marks and the Polycomb protein EZH2 indicate that *Bdnf* promoters I, II, IV and VI are bivalent in adult mice: (**b**) H3K27Me3, (**c**) EZH2, (**d**) JMJD3, (**e**) H3K4Me3 and (**f**) H3K27Ac. The levels of these proteins at promoter regions of the control genes *βActin* and *hoxA1* are also shown in the plots. (**g**) ChIP-qPCR analyses show that NMDA stimulation leads to H3K27Me3S28 phosphorylation and displacement of the polycomb protein EZH2, JMJD3 demethylase recruitment, H3K27Me3 demethylation and increased levels of the activator H3K4Me3 at the four *Bdnf* promoters I, II, IV and VI. H3K27 acetylation was observed only at promoters II and VI suggesting that this mark is responsible for the fast induction of these two promoters. (**h**) ChIP-qPCR analyses at promoter regions of the control genes *βActin* and *hoxA1* after NMDA stimulation. The data show that, except for the increased levels of JMJD3 observed at the *βActin* promoter, none of the other marks present changes after NMDA stimulation. Data are represented as mean±s.e.m. Statistical analysis by Mann–Whitney U-test (the value inside the bars indicates the number of animals used in independent experiments; *P<0.05; **P<0.01, ***P<0.001; for statistical analysis see Supplementary Table 4).

It has been described that H3K27Me3 phosphorylation at Serine 28 masks the recognition of H3K27Me3 by different specific antibodies^31^,^32^, an event also observed by us (see Supplementary Fig. 4A). It means that the decreases observed in the levels of H3K27Me3 at *Bdnf* promoters after stimulation could be attributed to epitope masking rather than true demethylation. However, experiments based on extracts obtained from neuronal cultures treated with alkaline phosphatase to remove the phosphate group from H3K27Me3S28p (see Supplementary Fig. 4B), followed by ChIP with H3K27Me3 antibodies, still revealed demethylation. These ChIPs show a significant decrease of H3K27Me3 at promoters IV and VI in phosphatase-treated samples indicating that H3K27Me3 demethylation does occur in an epitope unmasked situation (Supplementary Fig. 4C). *In vitro* assays using the human recombinant catalytic domain of JMJD3, suggest that JMJD3 is able to demethylate the phosphorylated peptide H3K27Me3S28p (Supplementary Fig. 5A–C). If this is the case *in vivo*, in this three-way scenario where H3K27Me3S28 phosphorylation, H3K27Me3 demethylation and H3K27Me3S28p demethylation occur, it becomes very difficult to assess the magnitude of the contribution of epitope masking to the H3K27Me3 decrease. In any case, our observations indicate that epitope masking would not be the major determinant of decreased H3K27Me3 levels under LTD because: (i) H3K27Me3 demethylation is prevented by inhibiting demethylase activity with GSK-J4 or by JMJD3 knockdown (Fig. 3e and Supplementary Fig. 3F); (ii) H3K27Me3 demethylation is prevented by inhibiting JMJD3 recruitment to chromatin (Fig. 3j,k); (iii) H3K27Me3 demethylation is still observed after LTD in samples treated with Alkaline Phosphatase, that is, in the absence of epitope masking (Supplementary Fig. 4C); and (iv) there is not a direct correlation between H3K27Me3S28 phosphorylation and H3K27Me3 decrease (see *Bdnf* promoters II and IV in Fig. 2f and *Bdnf* promoter I in Fig. 4g).

### *Bdnf* induction requires CREB-p/CBP/ JMJD3

Several signalling pathways lead to the activation of cyclic AMP response element-binding protein (CREB) kinases, which phosphorylate CREB on the residue Ser133 (refs 38, 39, 40). Upon phosphorylation at Ser133, CREB translocates to the nucleus and recruits the transcriptional co-activator CREB-binding protein (CBP) with histone acetyl transferase activity^41^. We speculated that a similar mechanism could explain the H3K27 acetylation induced by NMDA stimulation (see Fig. 2f). Consistent with this possibility, NMDA-LTD triggers a transient CREB phosphorylation at Serine 133 (CREB-pS133) 10 min after stimulation (Supplementary Fig. 6A). To determine the pathway by which NMDA stimulation, that is, Ca^2+^/calmodulin-dependent protein kinase II (CamKII) or protein kinase C (PKC), leads to CREB phopsphorylation, hippocampal neurons were treated with the CamKII inhibitor KN93 or the PKC inhibitor Chelerytrine before NMDA stimulation. The levels of pS133-CREB were then determined by western blot at 10 min after LTD induction. Either KN93 or chelerythrine were able to block CREB phosphorylation at 10 min after NMDA stimulation (Supplementary Fig. 6B and C), suggesting that NMDA-LTD may require synergic activities between PKC and CamKII, like previously reported during LTP. In fact, it was shown that PKC potentiates NMDA receptor gating, in turn enhancing Ca^2+^ influx and intracellular Ca^2+^/camodulin, which could trigger CaMKII autophosphorylation^42^,^43^,^44^. To further dissect the pathway leading to S133-CREB phosphorylation, we analysed the contribution of MSK1, a downstream kinase common to PKC and CamKII pathways. Hippocampal neurons were incubated with the MSK1 inhibitor H89 before NMDA stimulation. As shown in Supplementary Figure 6D, the treatment with H89-blocked CREB phosphorylation at 10 min after NMDA stimulation indicating that MSK1 triggers CREB phosphorylation at Ser 133. We can conclude that NMDA- LTD leads to S133-CREB phosphorylation *via* CamKII-PKC/MSK1 kinases. However, since H89 also inhibits PKA with a similar potency^45^, we could not exclude a role of PKA in the phosphorylation of CREB by NMDA.

Coherent with the CREB activation, NMDA triggers a transient and a significant increase in the acetylation of H3K27 at 10 min after treatment (Supplementary Fig. 6E). Such increased acetylation of H3 was not observed at other lysine residues, H3K9, H3K14 or H3K18, after NMDA addition (Supplementary Fig. 6F–H). To directly assess the functional association between H3K27 acetylation and CREB activation, we treated hippocampal neurons with CREB-pS133/CBP interaction inhibitor (CCIIh) to impair CREB-pS133/CBP binding^46^,^47^. This treatment blocked the transcriptional activation of promoters II and VI and impaired H3K27 acetylation at these promoters (Fig. 3f,g). These results confirm that CREB-p/CBP is required for H3K27 acetylation and activation of promoters II and VI by NMDA-LTD. No changes of H3K27Ac were observed at promoters I and IV at 10 min after induction and in fact, the activation of these promoters was unaffected by the inhibitor (Fig. 3f,g). Active CREB was found at the *Bdnf* promoters either in the presence or absence of the inhibitor in NMDA-stimulated neurons (Fig. 3h). However, treatment with the CREB/CBP inhibitor CCIIh blocked the recruitment of CBP (Fig. 3i).

The CCIIh inhibitor blocks the interaction between the KID domain of CREB and the KIX domain of CBP. This KIX domain in CBP also interacts with the p65 subunit of the transcriptional activator nuclear factor κB (NFκB), implying that the interaction CBP-p65 is also disrupted by CCIIh (ref. 46). Although a role of NFκB has been described in the activation of *Bdnf* promoters I and IV but not in the activation of promoters II and VI (refs 48, 49, 50), the possibility exists that the observed effect of CCIIh could be due to inhibition of the recruitment of CBP by NFκB to promoters II and VI.

Zhong *et al*.^51^ have shown that the activation of p65 by phosphorylation at its serine 276 is strictly required for the interaction with CBP: in Supplementary Fig. 7, we show that application of 20 μM NMDA to hippocampal neurons in culture was not able to trigger phosphorylation of p65Ser273 at 10 min after stimulation, indicating that p65 is not involved in the recruitment of CBP to *Bdnf* promoters at least at this time point after NMDA stimulation.

The interaction of CBP with the demethylase UTX from the lysine-specific demethylase 6 (KDM6) family has been described^52^, opening the possibility that CREB-p/CBP complex would be also involved in the recruitment of the demethylase JMJD3 (also a member of the KDM6 family) to the *Bdnf* promoters after stimulation. By ChIP experiments we confirmed that the recruitment of JMJD3 to promoters II, IV and VI is impaired by CCIIh (Fig. 3j). In addition, CCIIh blocked the H3K27Me3 demethylation induced by NMDA at promoters IV and VI (Fig. 3k). Note that CCIIh treatment also resulted in significantly higher levels of H3K27Me3 at promoter II after stimulation (Fig. 3k). These results support our previous conclusion that H3K27Me3 demethylation is required for induction of promoter II (Fig. 3d), but it occurs at lower levels than at promoters IV and VI. The direct interaction CBP-JMJD3 was further confirmed by co-immunoprecipitation (Supplementary Fig. 8).

From these results we conclude that the mechanism of *Bdnf* induction triggered by NMDA involves CREB-p-mediated recruitment of CBP and JMJD3 to all *Bdnf* promoters, yet rapid transcription from promoters II and VI requires H3K27Me3 demethylation followed by H3K27 acetylation.

### Validation in acute hippocampal slices

Our results indicate that *Bdnf* promoters II and VI are subjected to EZH2 repression and that, after specific neuronal stimuli, the increased transcriptional activity is due to the release of the repressor EZH2 as a consequence of H3K27Me3Ser28 phosphorylation, H3K27Me3 demethylation and H3K27 acetylation. Because our data were obtained in neurons grown *in vitro*, we decided to test our model in neurons that had developed and matured in a physiological environment. Thus, we analysed the effect of NMDA-LTD on the expression and epigenetic remodelling at *Bdnf* promoters in hippocampal slices prepared from adult mice. Similar to what occurs *in vitro*, NMDA addition to hippocampal slices resulted in increased levels of mRNA species II and VI at 10 min, and in increased levels of the four species at 30 min after stimulation (Fig. 4a). Likewise *in vitro*, higher increases were observed for mRNAs transcribed from promoters II and VI (Fig. 4a). Also paralleling the data in cultures, the repressive H3K27Me3 and EZH2 marks were detected at these four *Bdnf* promoters in non-NMDA-stimulated slices (Fig. 4b,c). High amounts of JMJD3 demethylase were also found at these promoters (Fig. 4d), suggesting that transcriptional activity requires H3K27Me3 demethylation. As expected, the presence of activation marks H3K4Me3 and H3K27Ac were also observed at promoters I, II, IV and VI at the same proportions than in neuronal cultures (Fig. 4e,f). However in hippocampal slices, the amount of H3K27Ac found at promoter II was comparable to the levels found at the repressed gene *hoxA1*, suggesting a low basal transcriptional activity for this promoter.

The ChIP experiments performed in hippocampal slices revealed that LTD induces phosphorylation of H3K27Me3S28p and displacement of EZH2 not only at promoters II and VI but also at promoters I and IV (Fig. 4g). These results strengthen the conclusion previously obtained from *in vitro* data that phosphorylation of H3K27Me3S28 displaces EZH2 repressor from all the four promoters (see Fig. 3c). In hippocampal slices, H3K27Me3 demethylation was also evident at promoter II (Fig. 4g), explaining the effect of GSK-J4 on this promoter *in vitro* (see Fig. 3d). Interestingly, increases of the activation mark H3K4Me3 was observed for all the four promoters in hippocampal slices (Fig. 4g). Similar to *in vitro* grown hippocampal neurons, H3K27 acetylation in hippocampal slices was only evident at promoters II and VI (Fig. 4g), suggesting that H3K27 acetylation may be the relevant modulator for the fast induction of these two promoters after stimulation. Except for an increase in JMJD3 interaction with *βActin* promoter, for which we do not have an explanation, no other changes in the levels of these marks were observed at the promoters of the control genes (Fig. 4h).

All in all, the results obtained in hippocampal slices reinforce the notion that these four promoters of *Bdnf* are controlled by PRC2 repressor and suggest that fast (10 min) response of promoters II and VI would be due to H3K27 acetylation.

## Discussion

In this work, we show that neuronal stimulation with low doses of NMDA, a protocol widely used to induce LTD, triggers the fast transient activation of *Bdnf* promoters II and VI, whereas promoters I and IV present a slower response with a transcriptional activation remaining stable for longer time.

The biological significance of this alternative promoter usage could be explained from the works developed by Baj *et al*.^14^ These authors have shown that the transcripts containing the 5′exons II and VI are transported to distal dendrites whereas the transcripts containing 5′exons I and IV are required in the soma or in proximal dendrites. By use of overexpression and silencing of specific *Bdnf* mRNA isoforms, the authors found that dendritic BDNF (from exons II and VI) plays a role in secondary dendrites' plasticity in response to external stimuli. Furthermore, the expression of individual *Bdnf* splice variants was shown to be relevant for the spatially restricted activation of TrkB receptors: that is, overexpression of exons I or IV transcripts led to phosphorylation of 80–100% of TrkB receptors within the first 45 μm from the soma, whereas overexpressing exons II or VI *Bdnf* variants led to 80% TrkB phosphorylation at 80 μm from the soma and beyond. Thus, the spatial segregation of *Bdnf* transcripts enables this neurotrophic factor to differentially shape distinct dendritic compartments.

At first sight it would seem that a fast induction of a promoter localized more than 50 kb 5′ away from the *Bdnf* coding exon (exon IX) is not, physiologically speaking, the optimal situation. However the fast induction of promoter II in our model would have an important biological significance. Because the ultimate destination of *Bdnf* mRNAs is dictated by the presence of different 5′-untranslated region exons, it means that the fast induction of promoters II and VI at 10 min after LTD induction would be required to provide the stimulated cell with the adequate amounts of 5′exons II and VI-containing transcripts at the appropriate timing to be delivered to distal dendrites.

**Figure 5:**
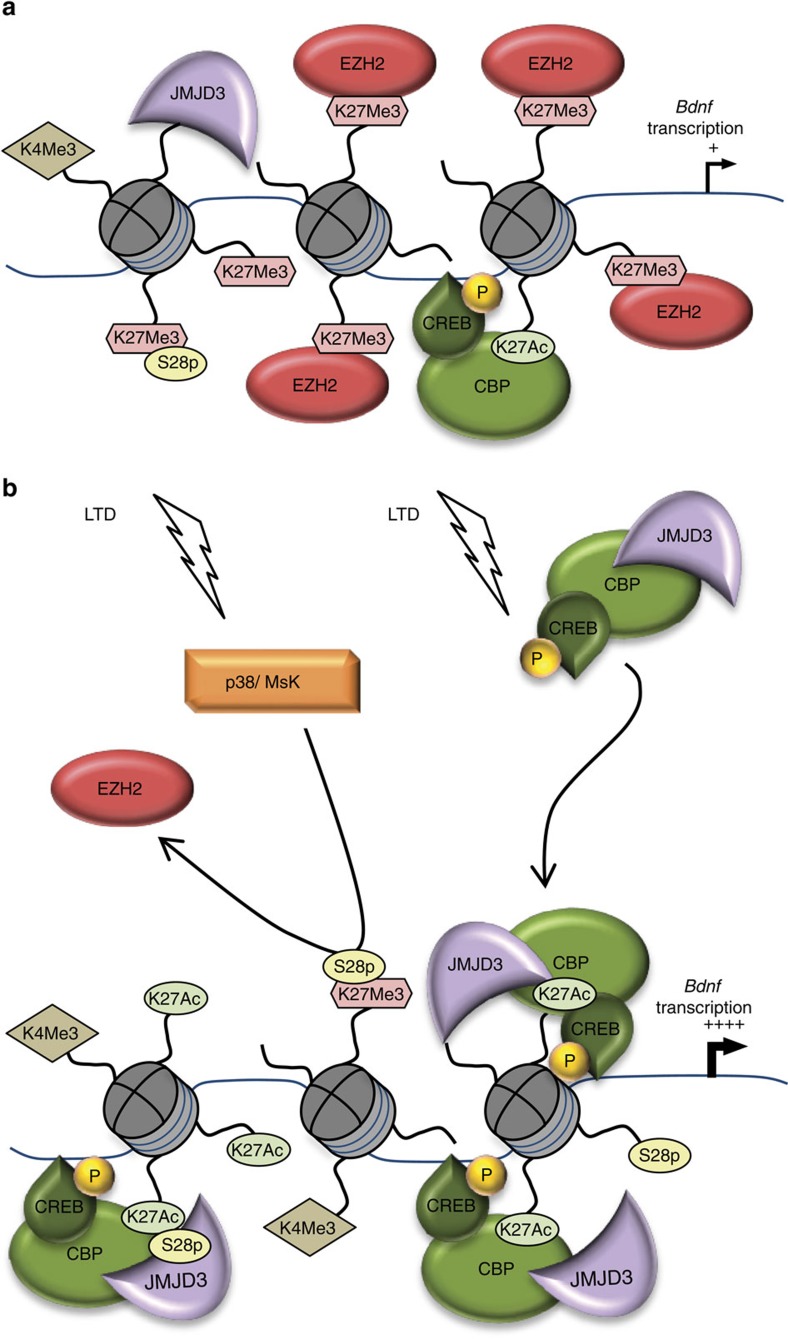
Schematic model describing the proposed mechanism for activation of *Bdnf* promoters. (**a**) In adult non-stimulated neurons, activator (H3K4Me3 and H3K27Ac) and repressor (H3K27Me3) marks coexist. *Bdnf* promoters are present in a partially repressed status exerted by EZH2. (**b**) LTD triggers the remodelling of the transcriptional complexes leading to increased levels of activator H3K4Me3 and recruitment of p38 MAPK/Msk1/2 that phosphorylate H3K27Me3 at its Ser 28 and displaces the repressor protein EZH2. LTD induction also leads to activation of CREB by phosphorylation at its Ser 133 and recruitment of the complex pSer133-CREB/CBP/JMJD3 to *Bdnf* promoters. The LTD stimulus triggers the increase of the activator mark H3K27Ac only at promoters II and VI, suggesting that H3K27 acetylation is an important factor in the high transient expression of these two promoters.

We have shown that, in adult non-stimulated neurons, *Bdnf* promoters are present in a partially repressed status exerted by EZH2. Application of NMDA at doses that produce chemical LTD leads to the remodelling of the transcriptional complexes, namely increased levels of activator H3K4Me3 and recruitment of H3K27Me3-demethylase JMJD3 and by the consequent decrease of the repressive mark H3K27Me3. We also found that LTD stimulus triggers the phosphorylation of the H3K27Me3 at its Ser 28 leading to the displacement of the repressor protein EZH2 (Fig. 5). In proof of functional association between these changes and activation of *Bdnf* expression, preventing H3K27Me3S28 phosphorylation blocks EZH2 release and the transcriptional stimulation of promoters II and VI.

It has been demonstrated that retinoic acid induce neural differentiation in human embryonic teratocarcinoma cells via histone H3K27Me3S28 phosphorylation, displacement of polycomb proteins and gene expression^31^. Our data suggest that a similar mechanism may operate in fully mature neurons *in vitro*, in response to a memory-related stimulus. Although H3K27Me3S28 phosphorylation, EZH2 derepression and H3K27Me3 demethylation also occur after LTD at *Bdnf* promoters I and IV, blocking these processes does not impair the late occurring transcriptional induction of these two promoters, suggesting that polycomb derepression is mostly restricted to promoters II and VI.

The molecular mechanism by which a physiological mnemonic process like LTD affects *Bdnf* expression has not been studied previously. However, it has been shown that methyl-CpG-binding protein 2 (MeCP2) represses promoter IV through the association with the co-repressor molecule Sin3a and histone deacetylase 1 (HDAC1)^53^,^54^,^55^,^56^. Membrane depolarization by KCl correlated with DNA demethylation and MeCP2 phosphorylation and dissociation from *Bdnf* promoter IV (refs 53, 55). NMDA receptor activation de-represses *Bdnf* promoters I and IV in correlation with reduced occupancy by HDAC1 and MeCP2 in cultured hippocampal neurons^49^,^57^. Since memory consolidation after fear conditioning involves LTD induction, it is possible that DNA demethylation and MeCP2 derepression would be also involved in the activation of promoters I and IV after LTD.

The chromodomain protein and transcription co-repressor chromodomain Y-like (CDYL) protein represses *Bdnf* promoter II in developing neurons by a polycomb-mediated mechanism. De-repression can be induced by electrical activity through the degradation of CDYL^58^. We analysed whether a CDYL-dependent mechanism would also contribute to the expression changes of any of the *Bdnf* transcripts triggered by NMDA. While CDYL was enriched at *Bdnf* promoter II this was not the case for promoters I, IV and VI (Supplementary Fig. 9A). The levels of CDYL bound to promoter II decreased significantly and remained low at 30 min after NMDA stimulation (Supplementary Fig. 9B) in coincidence with protein degradation induced by the stimulus (Supplementary Fig. 9C). However the basal levels of EZH2 are restored at promoter II, 30 min after induction (Supplementary Fig. 1A). These data indicate first, that CDYL would not be involved in the de-repression of *Bdnf* promoter VI and second, that CDYL would not be involved in EZH2 recruitment to promoter II at 30 min after stimulation. However it cannot be excluded that CDYL degradation would be required also in mature neurons together with H3K27Me3S28 phosphorylation for EZH2 displacement from promoter II.

The regulation of neuronal differentiation by members of the polycomb group of proteins has been widely described. In adult mice, a role for EZH2 in the regulation of adult neurogenesis has also been reported and impaired learning and memory was attributed to the decreased proliferation of the progenitor cells in *Ezh2* knockout mice^59^. We here show that EZH2 directly controls memory formation by regulating the expression of *Bdnf* gene in mature neurons.

LTD induction increases the levels of the activator mark H3K27Ac only at promoters II and VI, suggesting that H3K27 acetylation is an important factor in the high transient mRNA expression mediated by these two promoters (Fig. 5). It is well known that neuronal stimulation by NMDA activates a signalling cascade resulting in CREB phosphorylation^60^,^61^. Phosphorylated CREB moves to the nucleus and interacts with the histone acetyl transferase CBP. The presence of a CREB-binding site has been previously observed at promoter IV and a possible role for CREB in the regulation of this promoter has been proposed but not directly demonstrated^55^,^62^. Our results demonstrate that H3K27 acetylation is catalysed by the CREB-p/CBP complex, as inhibition of the CREB-p/CBP interaction impairs H3K27 acetylation and transcriptional activation of promoters II and VI. We also show that this mechanism is not involved in the activation of promoters I and IV.

Our results strengthen the notion that synaptic activity differentially controls the *Bdnf* promoters by use of epigenetic mechanisms that include H3K27 acetylation via CREB/CBP and polycomb de-repression in mature neurons. It is tempting to speculate that a similar epigenetic mechanism may regulate the expression of other early genes involved in learning and memory.

## Methods

### Primary hippocampal neurons

Primary cultures of hippocampal neurons were prepared from embryonic day 18 (E18) Wistar rats as described in Kaech and Banker^63^ For our experiments hippocampal neurons were kept in culture for 15 days *in vitro* (15 DIV), when they have reached full maturation.

Hippocampi were dissected and placed into ice-cold Hank's solution with 7 mM HEPES and 0.45% glucose. The tissue was then treated with 0.005% trypsin (trypsin 0.05% EDTA; (Invitrogen; Life Technologies Co.) and incubated at 37 °C for 16 min and then treated with DNase (72 μg ml^−1^; Sigma-Aldrich) for 1 min at 37 °C. Hippocampi were washed three times with Hank's solution. Cells were dissociated in 5 ml of plating medium (Minimum Essential Medium supplemented with 10% horse serum and 20% glucose) and cells were counted in a Neubauer Chamber. Cells were plated into dishes pre-coated with poly-D-lysine (Sigma-Aldrich) (750,000 cells in a 10 cm dish and 270,000 in a 6 cm dish) and placed into a humidified incubator containing 95% air and 5% CO_2_. The plating medium was replaced with equilibrated neurobasal media supplemented with B27 and GlutaMAX (Gibco; Life Technologies Co.). On DIV 7 the culture medium was replaced with medium without GlutaMAX. Cultures were used 15 DIV.

### Hippocampal Slices

Hippocampal slices were prepared from 7–9-months-old male C57BL/J mouse. Hippocampi were extracted in dissection solution (10 mM D-glucose, 4 mM KCl, 26 mM NaHCO3, 233.7 mM sucrose, 5 mM MgCl2 and 1:1,000 Phenol Red) and sliced in an automatic tissue chopper (McIlwain Tissue Chopper, Standard Table, 220 V, Ted Pella Inc.) to obtain 400 μm hippocampal slices. Then, slices were kept in artificial cerebrospinal fluid (119 mM NaCl, 2.5 mM KCl, 1 mM NaH_2_PO_4_, 11 mM glucose, 1.2 mM MgCl_2_, 2.5 mM CaCl2. Osmolarity was adjusted to 290 Osm) for 1 h. Finally the experiments were performed in artificial cerebrospinal fluid at 25 °C.

### Drug treatments

Stock solutions of CBP-CREB Interaction Inhibitor (250 mM (CCIIh); Millipore ref. 217505), KDM6A/B inhibitor (GSK-J4; 100 mM; Tocris Bioscience ref. 4594) and p-38 MAK inhibitor (SB203580; 100 mM; Axon Medchem ref. Axon 1363) were prepared in dimethyl sulfoxide (Sigma). NMDA (20 mM; Sigma-Aldrich ref. M3262) was prepared in Milli-Q water. Hippocampal neurons were treated with 5 μM CCIIh (1 h), 10 μM GSK-J4 (1 h) or 10μM SB203580 (1 h) before NMDA-LTD induction. LTD was induced by 20 μM NMDA for 5 min, and the medium was replaced. Samples were collected 5 or 25 min after LTD induction. Samples without LTD induction were used as a control.

### Quantitative RT–PCR

Total RNA from hippocampal neurons or slices were extracted with Trizol Reagent (Ambion/RNA Life Technologies Co.) following the manufacturer procedures and cleaned up using RNeasy Mini kit (Qiagen, Hilden, Germany). RNA was quantified by absorbance at 260 nm using a Nanodrop ND-100 (Thermoscientific; Themo Fisher Scientific Inc.). Retrotranscription to first strand cDNA was performed using RevertAid H Minus First Strand cDNA Synthesis kit (Thermoscientific; Themo Fisher Scientific Inc.). Briefly, 5 ng of synthesized cDNA was used to perform fast qPCR using GoTaq qPCR Master Mix (Promega Co., Madison, WI, USA) in ABI PRISM 7900HT SDS (Applied Biosystems; Life Technologies Co.) with the manufacturer's protocol. The primers purchased to Sigma-Aldrich (Supplementary Table 13) were used at 0.5 μM final concentration to detect *Bdnf* transcripts and *Jmjd3*. Three housekeeping genes *Gapdh*, *GusB* and *Pgk1* were used as endogenous controls.

### Chromatin immunoprecipitation

ChIP experiments were performed as described by Millanes-Romero *et al*.^64^ Briefly, 750,000 cells were crosslinked with 1% formaldehyde for 10 min at 37 °C. Slices were crosslinked with 1% formaldehyde for 15 min at room temperature. Crosslinking was stopped by adding glycine to a final concentration of 0.125 M for 2 min at room temperature. Samples were washed three times with cold PBS and homogenized in cold Soft Lysis Buffer (50 mM Tris (pH 8.0), 10 mM EDTA, 0.1% NP-40 and 10% glycerol) plus inhibitors (protease inhibitor (cOmplete, EDTA-free; Roche), phosphatase inhibitor cocktail 2 (Sigma-Aldrich) and NaBut (5 mM) (Sigma-Aldrich)). Finally, lysates were centrifuged at 3,000 r.p.m. at 4 °C for 15 min. Nuclei pellets were lysed with SDS Lysis Buffer (1% SDS, 10 mM EDTA and 50 mM Tris (pH 8.0)) plus inhibitors and extracts were sonicated with Bioruptor Plus 300 to generate 400–700 bp DNA fragments. Samples were centrifuged at 13,000 r.p.m., 4 °C, 10 min to remove insoluble material, and the supernatant containing DNA–protein complexes was collected. In addition, protein quantification from sonicated chromatin was performed using Pierce BCA protein assay kit (Thermoscientific; Themo Fisher Scientific Inc.). The chromatin was diluted 1/10 with dilution buffer (0,01% SDS, 1,1% Triton X-100, 1,2 mM EDTA pH8, 16,7 mM Tris pH 8 and 167 mM NaCl) and pre-cleared with 50 μl protein A/G agarose beads (Santa Cruz Biotechnology, Inc.) and 40 μg of normal IgG (Santa Cruz Biotechnology, Inc.) for each 500 μg of protein. Samples were placed in a rotor for 1–3 h at 4 °C. The mixture was centrifuged and the supernatant was collected. A total of 100 μg of protein were used for each ChIP assay, reserving 10 μg as the input. The antibodies (Supplementary Table 14) were added to the chromatin lysate, incubated on a rotor O/N at 4 °C. Immune complexes were precipitated by the addition protein A/G agarose beads. As a negative control, non-immune rabbit IgG (Santa Cruz Biotechnology, Inc.) was used in place of specific antibodies. Immunoprecipitated complexes were washed three times with the following buffers: Low-salt wash buffer (0,1% SDS, 1% Triton X-100, 2 mM EDTA pH8, 20 mM Tris pH 8 and 150 mM NaCl); high-salt wash buffer (0,1% SDS, 1% Triton X-100, 2 mM EDTA pH8, 20 mM Tris pH 8 and 500 mM NaCl); and LiCl wash buffer (250 mM LiCl, 1% NP-40, 1% NaDOC, 1 mM EDTA and 10 mM Tris pH8). Immune complexes were eluted in 100 μl of 1% SDS and 100 mM NaHCO_3_ at 37 °C for 30 min. DNA–protein crosslinks were reversed by adding NaCl to a final concentration of 200 mM O/N at 60 °C. Protein digestion was preformed 1 h at 37 °C by adding Proteinase K 0.04 mg ml^−1^ (Promega ref. MC5005), 50 mM EDTA pH8 and 500 mM Tris pH6,5 at final concentration. Finally, DNA was purified with QIAquick Gel Extraction Kit following the manufacturer procedures (Qiagen, Hilden, Germany) and eluted in 140 μl DNA/RNAse free MilliQ water. A total of 4 μl of purified DNA was used to perform a qPCR, using the listed primers on Supplementary Table 13. In all the cases, the ChIPs for histone marks have been normalized for general H3.

### Statistical analysis

All values are presented as mean±s.e.m. Mann–Whitney U-test and Kruskal–Wallis followed by Mann–Whitney U-test multiple comparisons with Bonferroni adjustment were used for statistical analysis of the data using SPSS6 (SPSS6 Statistics, IBM). *P* values lower than 0.05 were considered significant.

The sample size was chosen by setting the values of type I error (α) and power to the statistically adequate values of 0.05 and 0.80, respectively. Samples were discarded when LTD did not increase the levels of *Bdnf* mRNAs at 30 min after induction.

In the figures asterisks indicate *P* values as follows: *<0.05; **<0.01; and ***<0.001. The exact *P* values are shown in the Supplementary Tables.

Because in the *in vitro* and *ex vivo* experiments, treatments were compared with control conditions, there was no blinding in the experimental design. In all the cases, the control and correspondent treated samples were processed simultaneously and were obtained from the same culture or from slices prepared from the same animal.

### Animal handling

All the experiments were performed in accordance with European Union guidelines (2010/63/UE) regarding the use of laboratory animals.

## Additional information

**How to cite this article**: Palomer, E. *et al*. Neuronal activity controls Bdnf expression via Polycomb de-repression and CREB/CBP/JMJD3 activation in mature neurons. *Nat. Commun.* 7:11081 doi: 10.1038/ncomms11081 (2016).

## Supplementary Material

Supplementary Information Supplementary Figures 1-11, Supplementary Tables 1-14, Supplementary Methods and Supplementary References
