# Supplementary material for: Neuronal activity controls Bdnf expression via Polycomb de-repression and CREB/CBP/JMJD3 activation in mature neurons
Source: Nat Commun. 2016 Mar 24;7:11081. doi: 10.1038/ncomms11081 (PMC4820842; doi:10.1038/ncomms11081)
Supplement: Supplementary Information — Supplementary Figures 1-11, Supplementary Tables 1-14, Supplementary Methods and Supplementary References [file ncomms11081-s1.pdf]

Supplementary Figure 1

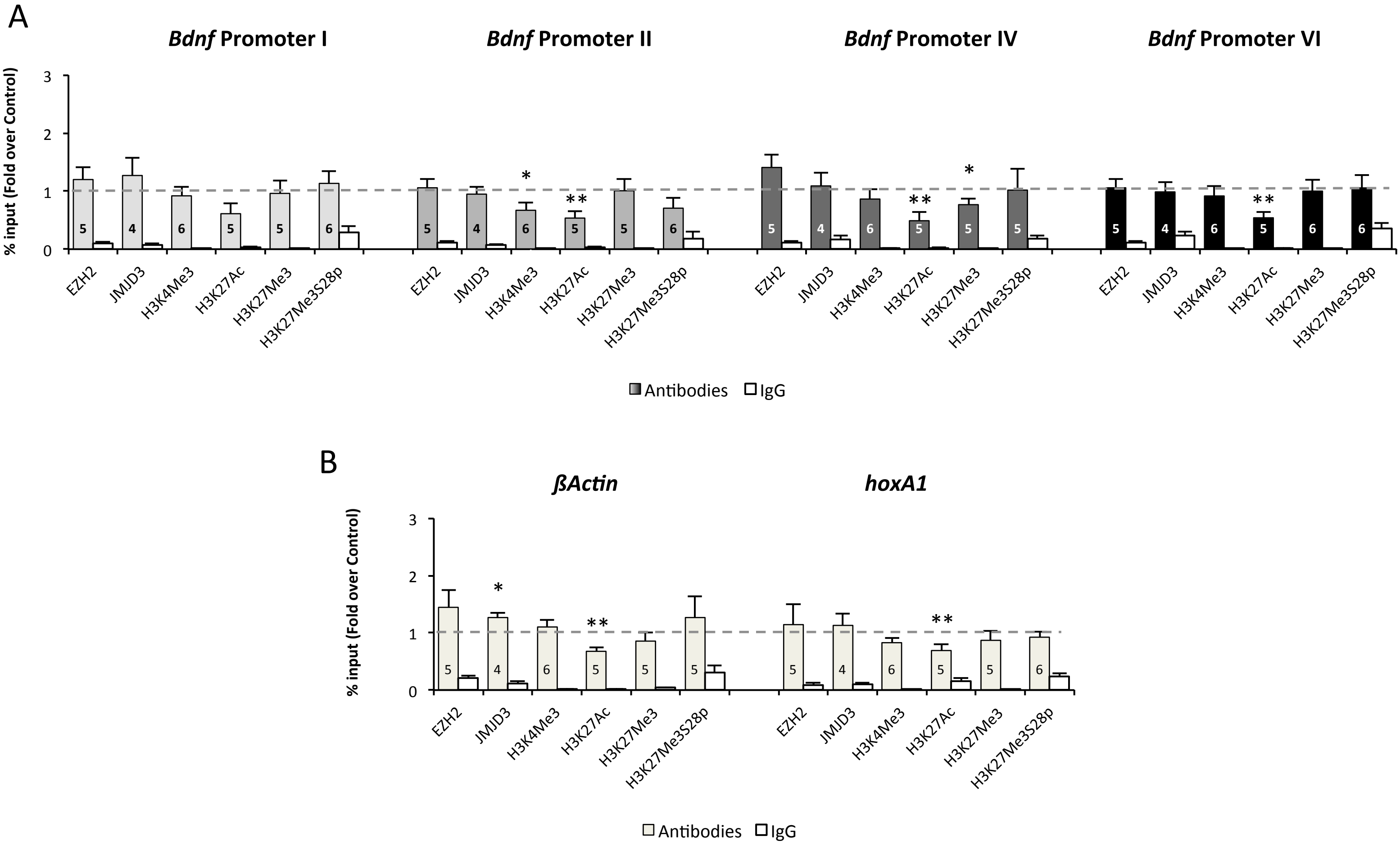

Supplementary Fig. 1. Basal epigenetic status is restored 30 min after stimulation.
A) ChIP-PCR analysis showing that the levels of most of the histone marks and regulatory proteins found at *Bdnf* promoters I, II, IV and VI return to basal levels at 30 min after NMDA stimulation. Differences with controls are still observed for H3K4Me3 at promoter II and H3K27Me3 at promoter IV. B) ChIP-qPCR analysis of these proteins 30 min after NMDA stimulation at *ßActin* and *hoxA1* control promoters. Levels of H3K27Ac that are lower than in non stimulated cells were found at all promoters suggesting a that H3K27 deacetylation could be a general effect observed at 30 min after NMDA addition. Data are represented as mean ± SEM. Statistical analysis by Mann-Whitney U test (number of independent experiments is indicated inside the bars; *p<0.05; **p<0.01). For statistical analysis see Supp. Table 5.

Supplementary Figure 2

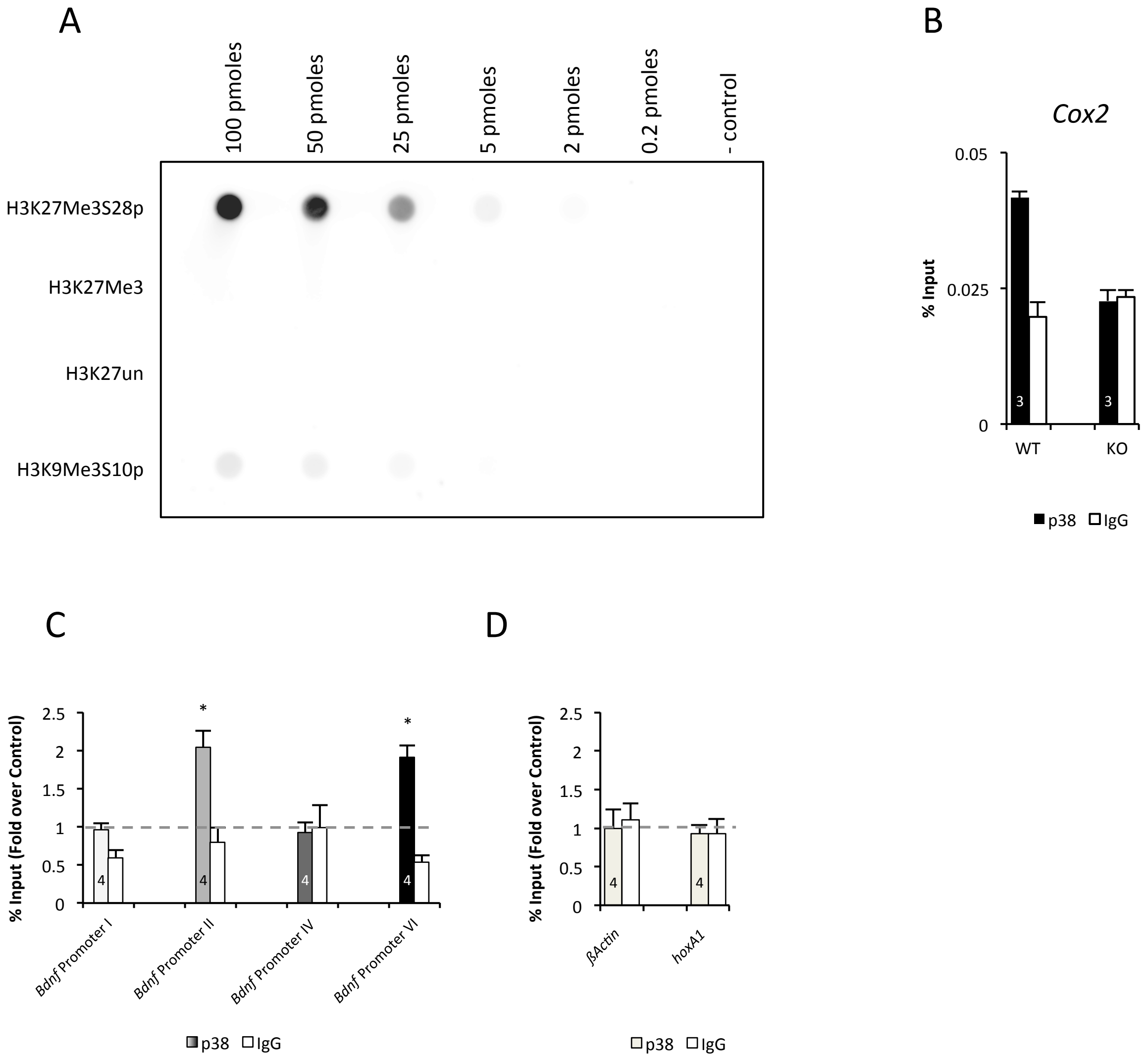

Supplementary Fig. 2. p38 is recruited to *Bdnf* promoters II and VI after LTD.
A) H3K27Me3S28p Rabbit antibody cross reactivity: Increasing concentrations of histone H3 peptides containing marks (H3K27Me3S28p, H3K27Me3, H3K9Me3S10p or unmodified H3) were loaded onto a Dot Blot and incubated wit the antibody against H3K27Me3S28p. The experiment shows a weak cross reactivity to H3K9Me3S10p. B) p38 antibody validation for ChIP: WT or p38-KO MEFs were treated with 100 mM NaCl for 45 min in order to induce p38 recruitment to *Cox2* promoter. The ChIP experiments show that p38 is detected, above of non specific signal, only in the WT cells. C) ChIP assays performed in neuronal cultures show that LTD induction triggers the recruitment of p38 to *Bdnf* promoters II and VI at 10 min after stimulation. D) p38 is not recruited to the promoter region of control genes *ßActin* and *hoxA1* after LTD induction. Data are represented as mean ± SEM. Statistical analysis by Mann-Whitney U test (number of independent experiments is indicated inside the bars; *p<0.05). For statistical analysis see Supp. Table 6.

Supplementary Figure 3

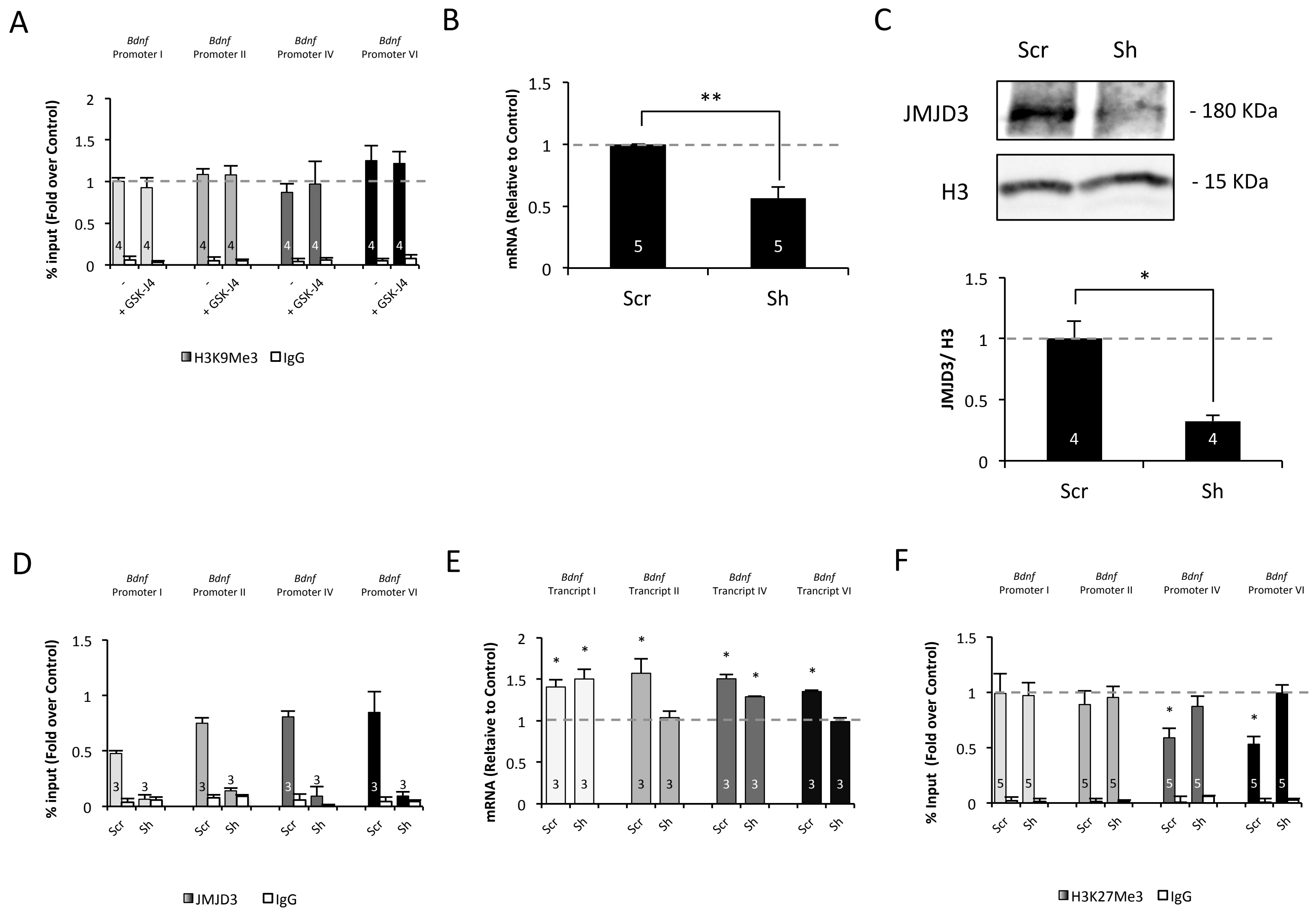

Supplementary Fig. 3. JMJD3 is involved in H3K27Me3 demethylation and *Bdnf* induction after LTD.
A) ChIP experiments showing that demethylation of the repressive mark H3K9Me3 is not involved in the transcriptional induction of *Bdnf* promoters I, II, IV and VI by NMDA-LTD. According to this conclusion, the treatment with the KDM inhibitor GSK-J4 has no effect on the levels of H3K9Me3 bound to these promoters in stimulated neurons. B) Quantification by qPCR show that the treatment of hippocampal neurons in culture with a lentivirus expressing a shRNA to knock down JMJD3 (Sh) led to a 50% reduction in the amount of the specific mRNA compared to cultures infected with leniviral particles expressing a scrambled shRNA (Scr). C) Western blot showing that JMJD3 knock down resulted in a 75% reduction of JMJD3 compared to scrambled-shRNA treated controls (the entire blot is shown in Supplementary Fig. 10). D) The ChIP experiments performed with the Abcam, ab38113 antibody show that the enrichment of JMJD3 at the *Bdnf* promoters is lost in cultures where JMJD3 was knocked down. E) The qPCR analysis show that JMJD3 knock down blocked the increase of the *Bdnf* transcripts II and VI triggered by LTD. F) The ChIP results show that the H3K27Me3 demethylation observed at promoters IV and VI in control neuronal cultures (Scr) at 10 min after NMDA addition was blocked by JMJD3 knock down (Sh). Data are represented as mean ± SEM. A) Statistical analysis by Kruskal-Wallis test and subsequent multiples comparisons by Mann-Whitney U test with Bonferroni adjustment. B-F) Statistical analysis by Mann-Whitney U test (number of independent experiments is indicated inside the bars; *p<0.05). For statistical analysis see Supp. Table 7.

Supplementary Figure 4

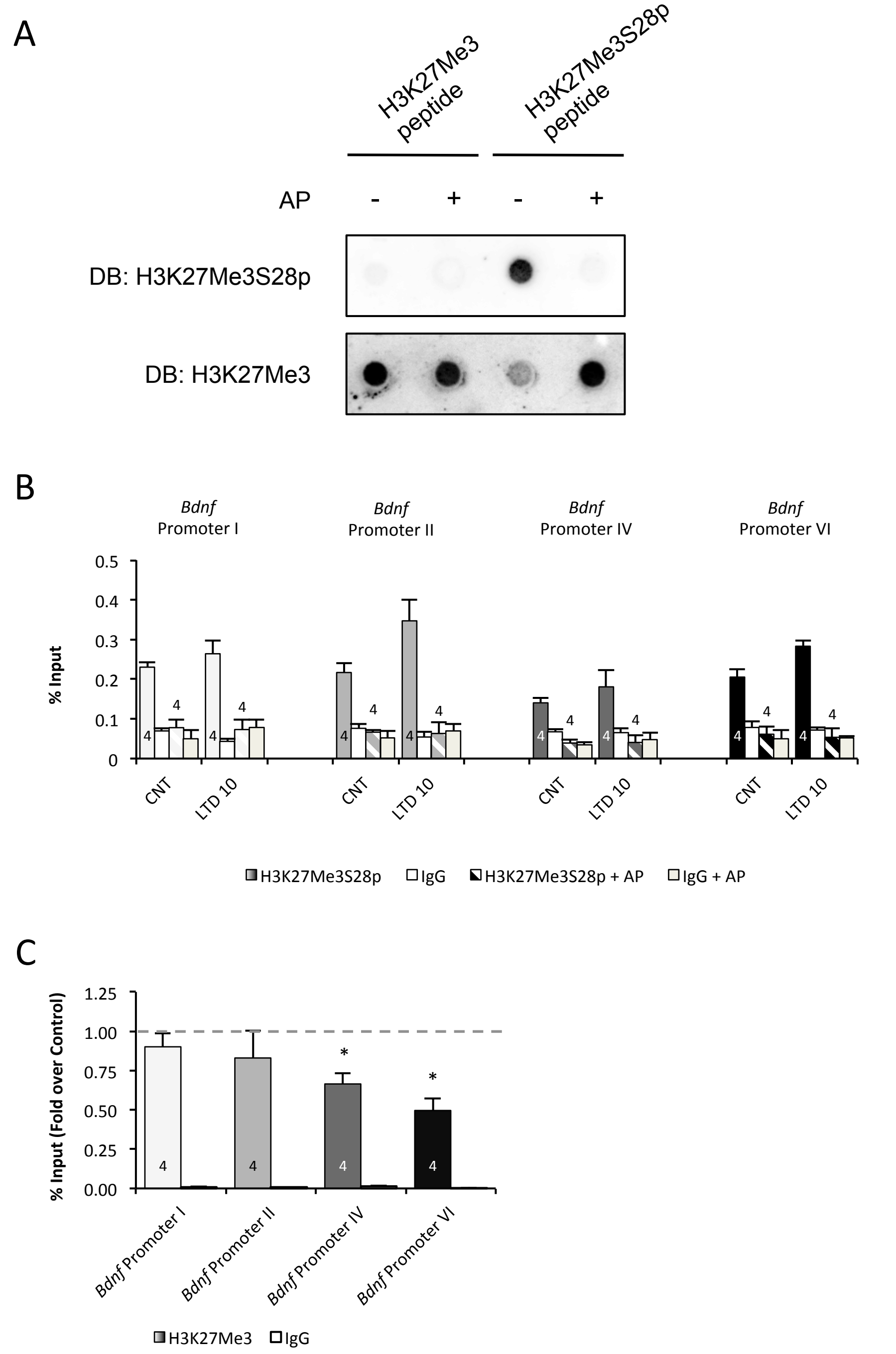

Supplementary Fig. 4. H3K27Me3 demethylation in the absence of epitope masking.
A) Dot blot showing that the antibody anti H3K27Me3 does not recognize the epitope in the presence of H3S28 phosphorylation. The peptides H3K27Me3 and H3K27Me3S28p were treated with alkaline phosphatase (AP) and then loaded onto a membrane. The dot blots were revealed with the specific antibodies. AP treatment to dephosphorylate H3S2p8 rescued de epitope recognition by the anti H3K27Me3 antibody. B) ChIP experiments showing that the treatment of the chromatin extracts with AP completely removed the phosphate groups from H3K27Me3S28p since only background levels (similar to levels obtained with IgG) of this mark were identified at the four *Bdnf* promoters treated. C) The ChIP experiments show that the levels of H3K27Me3 bound to promoters IV and VI decrease at 10 min after LTD induction in AP treated samples. Data are represented as mean ± SEM. Statistical analysis by Mann-Whitney U test (number of independent experiments in indicated inside the bars; *p<0.05). For statistical analysis see Supp. Table 8.

Supplementary Figure 5

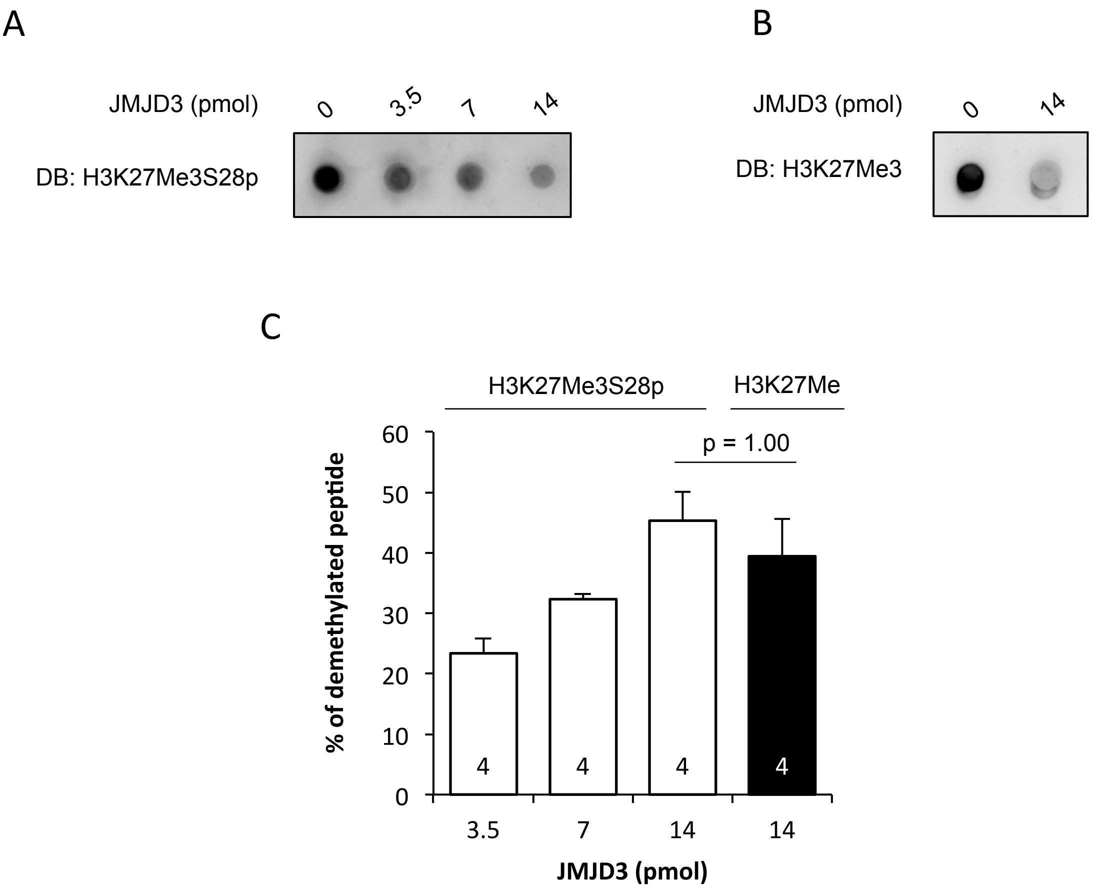

Supplementary Fig. 5. JMJD3 is able to demethylate H3K27Me3S28p *in vitro*.
A) Dot blot revealed for H3K27Me3S28p indicate that the recombinant JMJD3 catalytic domain is able to demethylate the phosphorylated peptide H3K27Me3S28p *in vitro*. B) Control demethylation recombinant JMJD3 catalytic domain assay on H3K27Me3 peptide. C) Quantification of dot blots as shown in panels A and B. Number of independent experiments is indicated inside the bars, for statistical analysis see Supp. Table 9.

Supplementary Figure 6

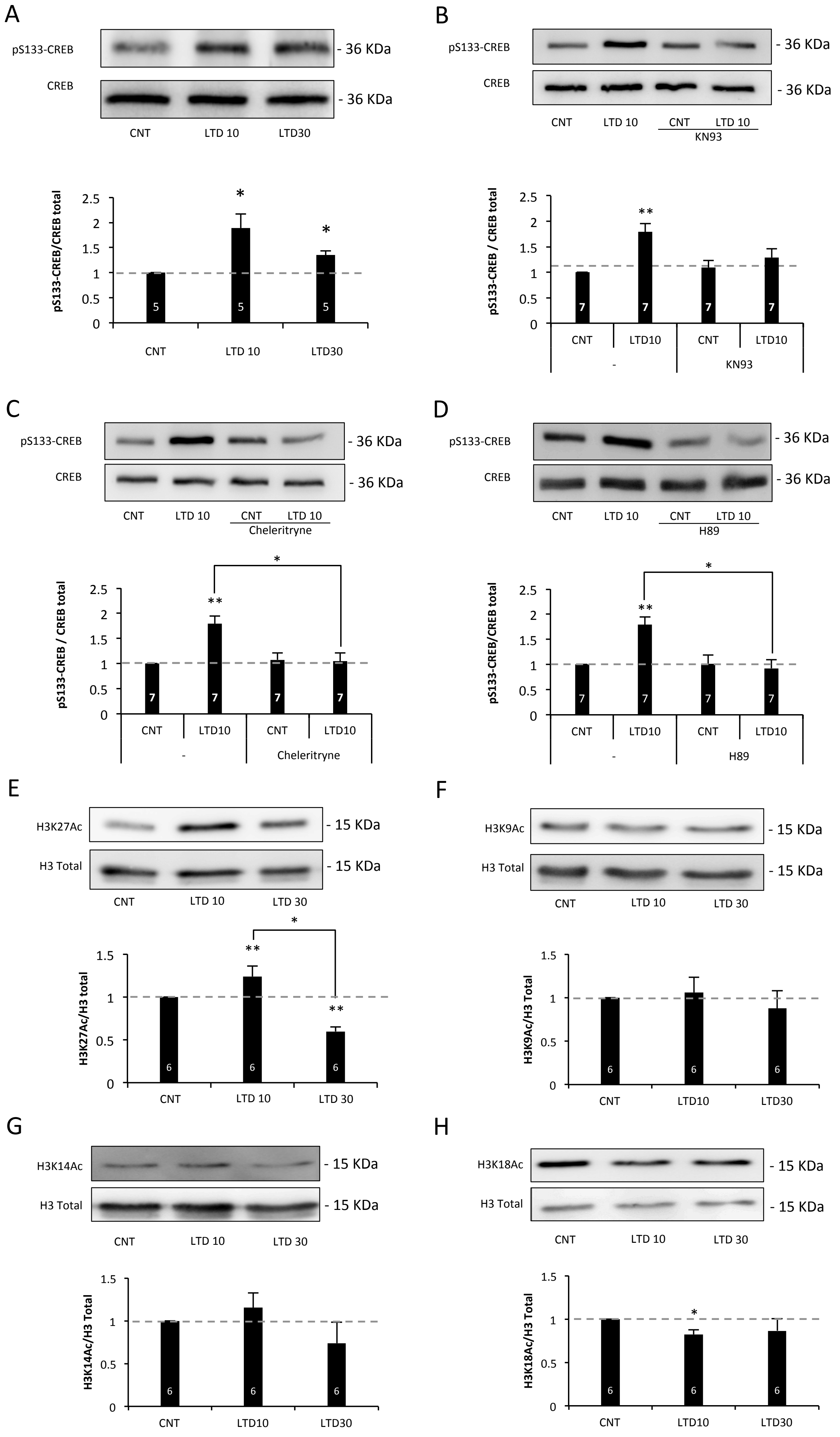

Supplementary Fig. 6A. NMDA stimulation triggers the transient CREB phosphorylation at Ser133 in mature hippocampal neurons.

Western blot studies show the CREB phosphorylation at its Ser133 in basal control conditions (CNT), at 10 min (LTD10) and at 30 min (LTD30) after NMDA stimulation. Data are represented as mean ± SEM. Statistical analysis by Kruskal-Wallis test and subsequent multiples comparisons by Mann-Whitney U test with Bonferroni adjustment (entire blot is shown in Supplementary Fig. 10; number of independent experiments is indicated inside the bars; *p<0.05; **p<0.01, ***p<0.001). For statistical analysis see Supp. Table 10.

Supplementary Fig. 6B-D: NMDA dependent LTD leads to S133-CREB phosphorylation via CamKII-PKC/MSK1 kinases. Western blots and it quantification showing that the S133-CREB phosphorylation observed in neuronal cultures at 10 min after LTD induction (LTD10) is blocked by treatment with the CamKII inhibitor KN93 (B), the PKC inhibitor Chelerytrine (C) and the Msk1 inhibitor H89 (D). Data are represented as mean ± SEM. Statistical analysis by Kruskal-Wallis test and subsequent multiples comparisons by Mann-Whitney U test with Bonferroni adjustment (entire blots are shown in Supplementary Fig. 10; number of independent experiments is indicated inside the bars; *p<0.05; **p<0.01). For statistical analysis see Supp. Table 10.

Supplementary Fig. 6E-H. NMDA stimulation triggers Histone H3 acetylation at Lysine 27, but not at Lysines 9, 14 or 18 in mature hippocampal neurons.

Western blots analysis of histone H3 acetylation at different Lysine residues in basal conditions, at 10 min and at 30 min after NMDA stimulation. The bar plots show the quantification of the western blots. Each acetylated form was corrected for total levels of H3 histone. Data are represented as mean ± SEM. Statistical analysis by Kruskal-Wallis test and subsequent multiples comparisons by Mann-Whitney U test with Bonferroni adjustment (entire blots are shown in Supplementary Figs. 10-11; number of independent experiments is indicated inside the bars; *p<0.05; **p<0.01). For statistical analysis see Supp. Table 10.

Supplementary Figure 7

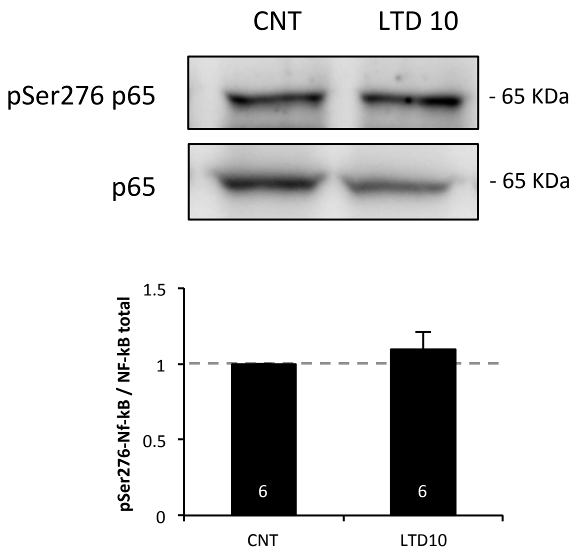

Supplementary Fig. 7. p65-Serine 276 is not phosphorylated at 10 min after NMDA-LTD.
Western blot and its quantification show that stimulation of hippocampal neurons in culture with 20μM NMDA is not able to trigger phosphorylation of p65Ser273 at 10 min after stimulation (LTD10). CNT: control non stimulated neurons. Data are represented as mean ± SEM. Statistical analysis by Mann-Whitney U test (entire blot is shown in Supplementary Fig. 11; number of independent experiments is indicated inside the bars). For statistical analysis see Supp. Table 11.

Supplementary Figure 8

A

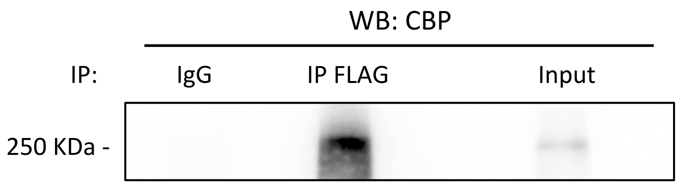

Supplementary Fig. 8. CBP coimmunoprecipitates with JMJD3.
Detection of CBP by Western blot in samples where JMJD3-FLAG or IgG (negative control) were immunoprecipitated from total protein extracts prepared from HEK transfected cells expressing both proteins. The coimmunoprecipitation of CBP and JMJD3 confirms the interaction of these proteins (entire blot is shown in Supplementary Fig. 11).

Supplementary Figure 9

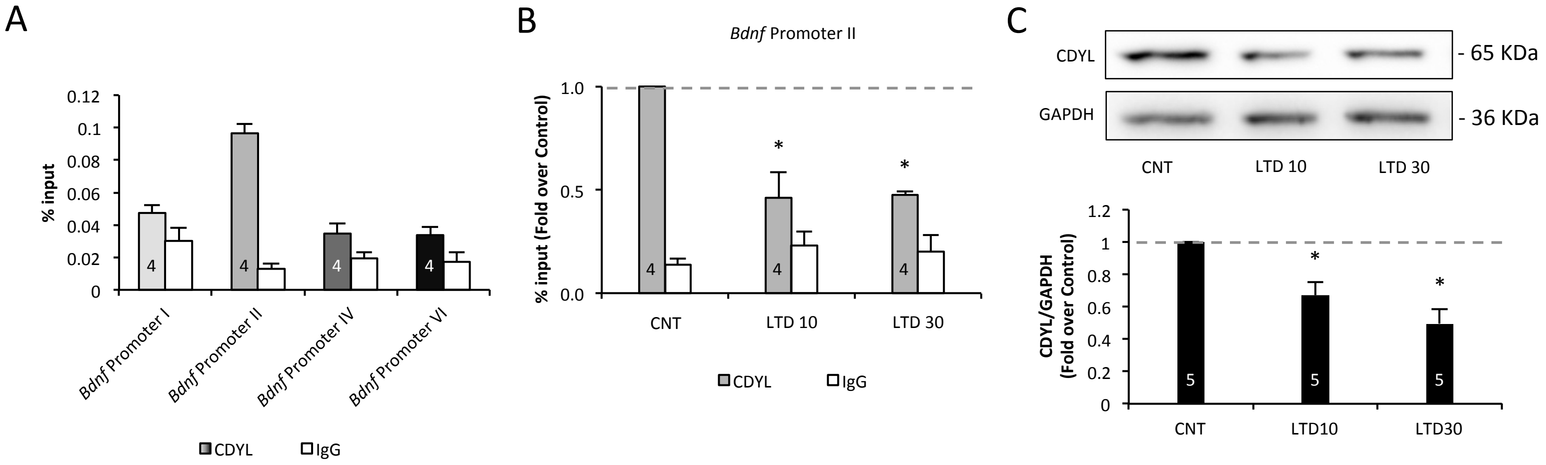

Supplementary Fig. 9. NMDA stimulation modulate CDYL attachment at *Bdnf* promoter II in mature hippocampal neurons.

A) ChIP analysis showing the presence of CDYL only at *Bdnf* promoter II. B) The ChIPs show that CDYL levels decrease at promoter II after NMDA stimulation, opening the possibility that CDYL-mediated de-repression of promoter II could be also playing a role in mature neurons. C) Western blot analysis and its cuantification show that NMDA stimulation induces CDYL degradation in mature neurons. Data are represented as mean ± SEM. Statistical analysis by Kruskal-Wallis test and subsequent multiples comparisons by Mann-Whitney U test with Bonferroni adjustment (entire blot is shown in Supplementary Fig. 11; number of independent experiments is indicated inside the bars; *p<0.05; **p<0.01, ***p<0.001). For statistical analysis see Supp. Table 12.

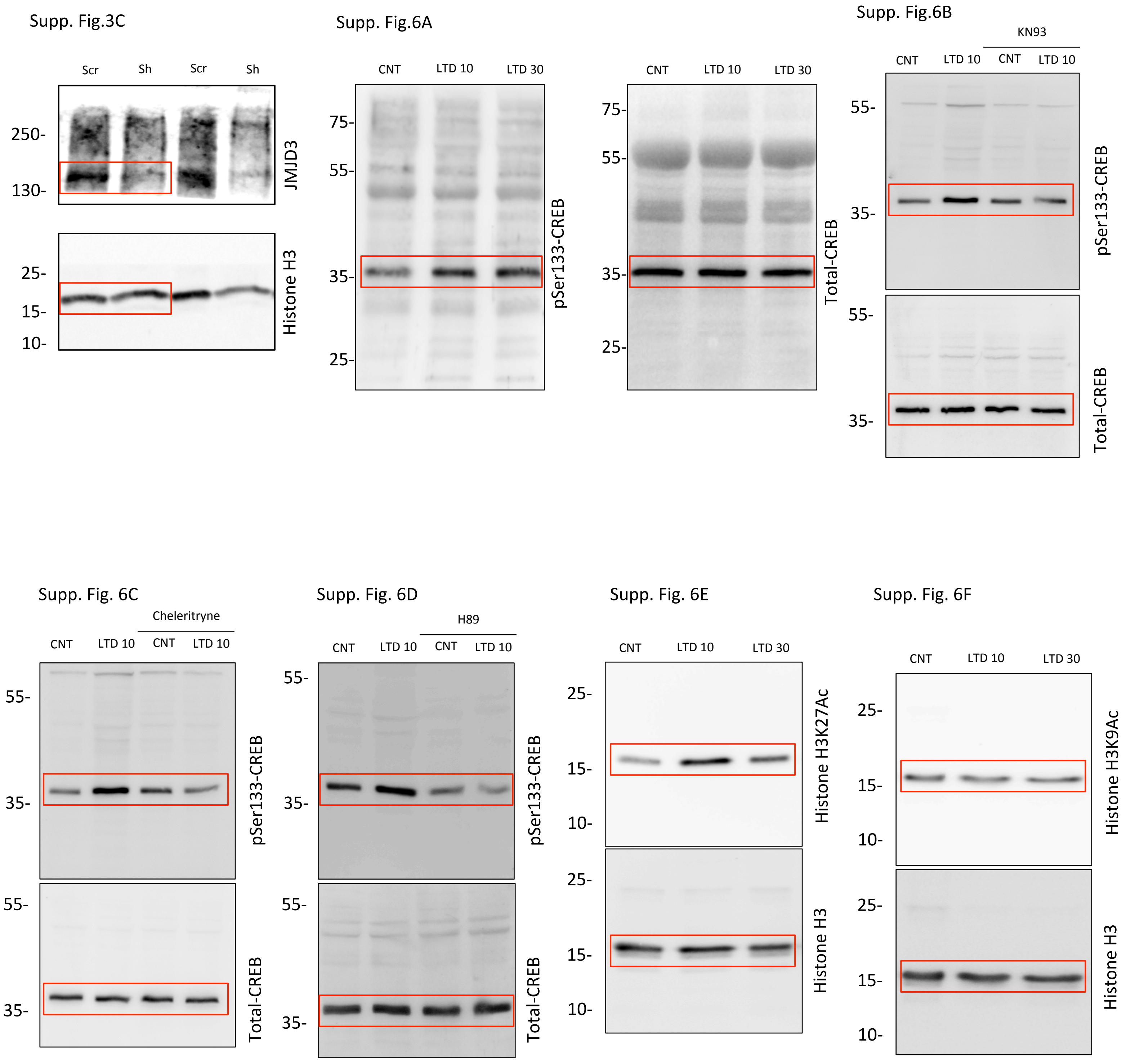

Supplementary Figure 10. Entire blots corresponding to the Figures shown in the paper.
The red rectangles indicate the portion of each blot used in the respective figures.

Supp. Fig. 6G

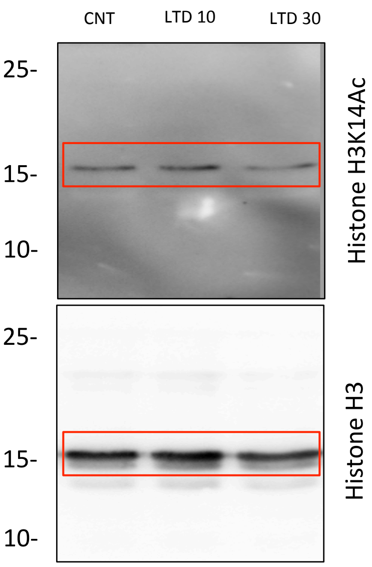

Supp. Fig. 6H

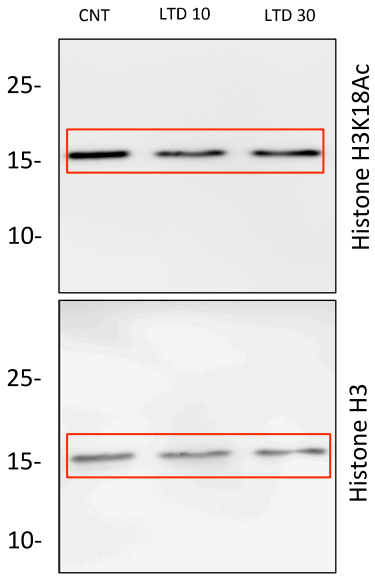

Supp. Fig. 7

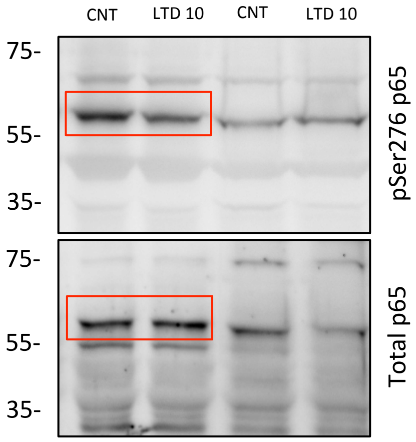

Supp. Fig. 8

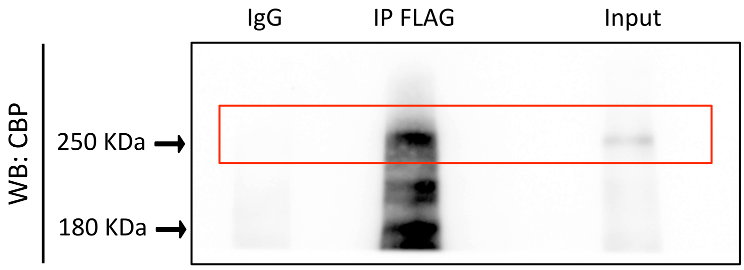

Supp. Fig. 9C

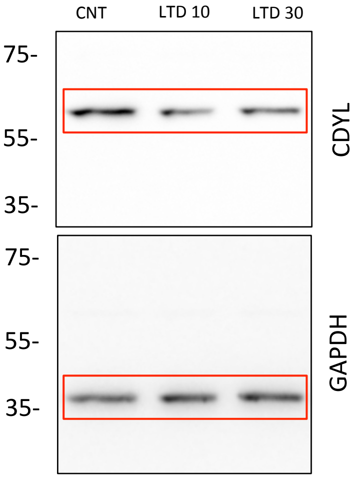

Supplementary Figure 11. Entire blots corresponding to the Figures shown in the paper.
The red rectangles indicate the portion of each blot used in the respective figures.

Supplementary Table 1: Data and statistics corresponding to Figure 1

A)

	min	Mean	N	SEM
Bdnf Transctipt I	0	1,00	13	0,00
	10	0,95	12	0,08
	30	1,34	13	0,12
	60	1,25	8	0,09
	90	1,27	9	0,10
	180	1,37	9	0,09

	min	Mean	N	SEM
Bdnf Transctipt II	0	1,00	13	0,00
	10	1,27	13	0,07
	30	2,07	12	0,23
	60	1,29	9	0,12
	90	1,22	9	0,13
	180	1,10	9	0,14

	min	Mean	N	SEM
Bdnf Transctipt IV	0	1,00	13	0,00
	10	1,01	12	0,08
	30	1,31	13	0,07
	60	1,36	9	0,06
	90	1,38	8	0,07
	180	1,14	9	0,06

	min	Mean	N	SEM
Bdnf Transctipt VI	0	1,00	14	0,00
	10	1,46	14	0,15
	30	2,13	14	0,13
	60	1,07	9	0,10
	90	1,00	9	0,07
	180	0,89	9	0,14

B)

Bonferroni adjustment	95% confidence interval	99% confidence interval	99.9% confidence interval
	(*) p < 1.67E-02	(**) p < 3.33E-03	(***) p < 3.33E-04

	min	Mean	N	SEM	Kruskal-Wallis Test	Mann-Whitney U Test Control / LTD 10	Mann-Whitney U Test Control / LTD 30	Mann-Whitney U Test LTD10 / LTD 30
Bdnf Transctipt I	0	1,00	13	0,00	2,31E-02	1,00E+00	1,04E-02	3,50E-02
	10	0,95	12	0,08				
	30	1,34	13	0,12				

	min	Mean	N	SEM	Kruskal-Wallis Test	Mann-Whitney U Test Control / LTD 10	Mann-Whitney U Test Control / LTD 30	Mann-Whitney U Test LTD10 / LTD 30
Bdnf Transctipt II	0	1,00	13	0,00	1,95E-07	3,64E-06	4,76E-06	1,10E-03
	10	1,27	13	0,07				
	30	2,07	12	0,23				

	min	Mean	N	SEM	Kruskal-Wallis Test	Mann-Whitney U Test Control / LTD 10	Mann-Whitney U Test Control / LTD 30	Mann-Whitney U Test LTD10 / LTD 30
Bdnf Transctipt VI	0	1,00	13	0,00	9,22E-05	1,27E-01	3,64E-06	4,68E-03
	10	1,01	12	0,08				
	30	1,31	13	0,07				

	min	Mean	N	SEM	Kruskal-Wallis Test	Mann-Whitney U Test Control / LTD 10	Mann-Whitney U Test Control / LTD 30	Mann-Whitney U Test LTD10 / LTD 30
Bdnf Transctipt VI	0	1,00	14	0,00	4,08E-06	5,96E-03	1,49E-06	2,82E-03
	10	1,46	14	0,15				
	30	2,13	14	0,13				

Supplementary Table 2: Data and statistics corresponding to Figure 2

A)

		Mean	N	SEM
H3K27Ac	*Bdnf* Promoter I	0,30	5	0,08
	Bdnf Promoter II	0,15	5	0,05
	Bdnf Promoter IV	0,36	5	0,07
	Bdnf Promoter VI	0,33	5	0,08
	ßActin	0,61	4	0,12
	hoxA1	0,02	4	0,01

		Mean	N	SEM
IgG	*Bdnf* Promoter I	0,045	5	0,018
	Bdnf Promoter II	0,025	5	0,016
	Bdnf Promoter IV	0,041	5	0,019
	Bdnf Promoter VI	0,008	5	0,003
	ßActin	0,006	4	0,003
	hoxA1	0,004	4	0,002

B)

		Mean	N	SEM
H3K4Me3	*Bdnf* Promoter I	1,78	5	0,27
	Bdnf Promoter II	0,66	5	0,22
	Bdnf Promoter IV	0,86	5	0,21
	Bdnf Promoter VI	1,16	5	0,38
	ßActin	1,80	4	0,10
	hoxA1	0,62	4	0,03

		Mean	N	SEM
IgG	*Bdnf* Promoter I	0,045	5	0,018
	Bdnf Promoter II	0,025	5	0,016
	Bdnf Promoter IV	0,041	5	0,019
	Bdnf Promoter VI	0,008	5	0,003
	ßActin	0,006	4	0,003
	hoxA1	0,004	4	0,002

C)

		Mean	N	SEM
H3K27Me3	*Bdnf* Promoter I	1,69	5	0,63
	Bdnf Promoter II	2,61	5	0,89
	Bdnf Promoter IV	1,61	5	0,36
	Bdnf Promoter VI	2,53	5	0,81
	ßActin	0,21	4	0,19
	hoxA1	4,39	4	1,05

		Mean	N	SEM
IgG	*Bdnf* Promoter I	0,045	5	0,018
	Bdnf Promoter II	0,025	5	0,016
	Bdnf Promoter IV	0,041	5	0,019
	Bdnf Promoter VI	0,008	5	0,003
	ßActin	0,006	4	0,003
	hoxA1	0,004	4	0,002

D)

		Mean	N	SEM
EZH2	*Bdnf* Promoter I	2,05	4	0,30
	Bdnf Promoter II	2,47	4	0,44
	Bdnf Promoter IV	1,78	4	0,25
	Bdnf Promoter VI	2,01	4	0,27
	ßActin	0,25	4	0,08
	hoxA1	2,08	4	0,26

		Mean	N	SEM
IgG	*Bdnf* Promoter I	0,008	4	0,001
	Bdnf Promoter II	0,013	4	0,003
	Bdnf Promoter IV	0,008	4	0,002
	Bdnf Promoter VI	0,012	4	0,003
	ßActin	0,014	4	0,005
	hoxA1	0,010	4	0,002

E)

		Mean	N	SEM
JMJD3	*Bdnf* Promoter I	0,41	4	0,11
	Bdnf Promoter II	0,54	4	0,14
	Bdnf Promoter IV	0,41	4	0,14
	Bdnf Promoter VI	0,40	4	0,12
	ßActin	0,52	4	0,12
	hoxA1	0,05	4	0,02

		Mean	N	SEM
IgG	*Bdnf* Promoter I	0,026	4	0,015
	Bdnf Promoter II	0,031	4	0,024
	Bdnf Promoter IV	0,040	4	0,001
	Bdnf Promoter VI	0,040	4	0,032
	ßActin	0,038	4	0,028
	hoxA1	0,022	4	0,013

F)

		Mean	N	SEM	Mann-Whitney U Test
Bdnf Promoter I	EZH2	0,91	5	0,13	5,77E-01
	JMJD3	1,23	4	0,26	2,19E-01
	H3K4Me3	1,02	7	0,13	1,52E-01
	H3K27Ac	0,91	7	0,14	6,33E-01
	H3K27Me3	1,00	7	0,14	1,52E-01
	H3K27Me3S28p	1,34	7	0,30	1,52E-01

			Mean	N	SEM
Bdnf Promoter I	EZH2	IgG	0,126	5	0,053
	JMJD3	IgG	0,083	4	0,012
	H3K4Me3	IgG	0,003	7	0,001
	H3K27Ac	IgG	0,030	7	0,010
	H3K27Me3	IgG	0,007	7	0,003
	H3K27Me3S28p	IgG	0,165	7	0,058

		Mean	N	SEM	Mann-Whitney U Test
Bdnf Promoter II	EZH2	0,54	6	0,12	3,35E-03
	JMJD3	1,47	4	0,06	1,19E-02
	H3K4Me3	1,41	6	0,11	2,09E-03
	H3K27Ac	1,36	6	0,06	1,53E-03
	H3K27Me3	1,15	7	0,16	6,33E-01
	H3K27Me3S28p	2,10	6	0,53	1,11E-03

			Mean	N	SEM
Bdnf Promoter II	EZH2	IgG	0,123	6	0,050
	JMJD3	IgG	0,091	4	0,023
	H3K4Me3	IgG	0,005	6	0,003
	H3K27Ac	IgG	0,070	6	0,033
	H3K27Me3	IgG	0,014	7	0,010
	H3K27Me3S28p	IgG	0,206	6	0,070

		Mean	N	SEM	Mann-Whitney U Test
Bdnf Promoter IV	EZH2	0,57	6	0,03	2,09E-03
	JMJD3	1,80	4	0,24	1,19E-02
	H3K4Me3	0,95	7	0,20	1,52E-01
	H3K27Ac	0,86	7	0,07	1,19E-02
	H3K27Me3	0,87	6	0,05	6,01E-04
	H3K27Me3S28p	1,11	7	0,26	6,33E-01

			Mean	N	SEM
Bdnf Promoter IV	EZH2	IgG	0,091	6	0,032
	JMJD3	IgG	0,107	4	0,023
	H3K4Me3	IgG	0,005	7	0,002
	H3K27Ac	IgG	0,040	7	0,014
	H3K27Me3	IgG	0,008	6	0,004
	H3K27Me3S28p	IgG	0,229	7	0,076

		Mean	N	SEM	Mann-Whitney U Test
Bdnf Promoter VI	EZH2	0,54	6	0,12	3,35E-03
	JMJD3	1,55	4	0,10	1,19E-02
	H3K4Me3	1,38	6	0,03	2,82E-03
	H3K27Ac	1,50	6	0,18	2,09E-03
	H3K27Me3	0,59	6	0,04	1,11E-03
	H3K27Me3S28p	1,77	6	0,26	1,11E-03

			Mean	N	SEM
Bdnf Promoter VI	EZH2	IgG	0,066	6	0,017
	JMJD3	IgG	0,083	4	0,011
	H3K4Me3	IgG	0,003	6	0,001
	H3K27Ac	IgG	0,036	6	0,010
	H3K27Me3	IgG	0,006	6	0,004
	H3K27Me3S28p	IgG	0,254	6	0,054

G)

		Mean	N	SEM	Mann-Whitney U Test
ßActin	EZH2	1,10	5	0,17	9,47E-02
	JMJD3	1,34	4	0,20	2,19E-01
	H3K4Me3	1,10	7	0,17	6,33E-01
	H3K27Ac	0,96	7	0,08	6,33E-01
	H3K27Me3	1,07	7	0,24	6,33E-01
	H3K27Me3S28p	1,14	6	0,13	2,77E-01

			Mean	N	SEM
ßActin	EZH2	IgG	0,269	5	0,104
	JMJD3	IgG	0,070	4	0,007
	H3K4me3	IgG	0,006	7	0,002
	H3K27Ac	IgG	0,016	7	0,006
	H3K27me3	IgG	0,052	7	0,012
	H3K27me3S28p	IgG	0,397	6	0,072

		Mean	N	SEM	Mann-Whitney U Test
hoxA1	EZH2	1,14	5	0,36	5,50E-01
	JMJD3	1,12	4	0,21	2,19E-01
	H3K4Me3	0,83	6	0,08	1,00E+00
	H3K27Ac	0,69	7	0,10	6,33E-01
	H3K27Me3	0,87	7	0,17	6,33E-01
	H3K27Me3S28p	0,92	7	0,10	6,33E-01

			Mean	N	SEM
hoxA1	EZH2	IgG	0,071	5	0,018
	JMJD3	IgG	0,061	4	0,023
	H3K4me3	IgG	0,002	6	0,001
	H3K27Ac	IgG	0,131	7	0,050
	H3K27me3	IgG	0,003	7	0,002
	H3K27me3S28p	IgG	0,097	7	0,029

Supplementary Table 3: Data and statistics corresponding to Figure 3

A)

Bonferroni adjustment: 95% confidence interval (*) p < 1.25E-02; 99% confidence interval (**) p < 2.50E-03; 99,9% confidence interval (***) p < 2.50E-04

		SB-203580	Mean	N	SEM	Kruskal-Wallis Test	Mann-Whitney U Test Control/LTD30	Control/Control +	Control/LTD30+	LTD30/Control+	LTD30/LTD30+	Control+/LTD30+
Bdnf Transcript I	Control	-	1,00	13	0,00	5,04E-02	1,04E-02	2,72E-01	3,14E-03	1,44E-01	6,22E-01	2,59E-01
	LTD 30	-	1,34	13	0,13							
	Control	+	1,11	12	0,08							
	LTD 30	+	1,32	12	0,12							
Bdnf Transcript II	Control	-	1,00	13	0,00	1,01E-05	4,76E-06	8,21E-04	1,00E+00	2,47E-05	6,02E-05	2,51E-01
	LTD 30	-	2,07	12	0,15							
	Control	+	0,71	14	0,08							
	LTD 30	+	0,89	14	0,11							
Bdnf Transcript IV	Control	-	1,00	13	0,00	1,42E-03	3,64E-06	1,81E-01	5,95E-05	7,26E-02	6,98E-01	8,08E-02
	LTD 30	-	1,31	13	0,06							
	Control	+	1,19	14	0,08							
	LTD 30	+	1,53	14	0,14							
Bdnf Transcript VI	Control	-	1,00	14	0,00	4,52E-02	1,49E-06	3,49E-02	6,73E-01	2,49E-05	1,27E-04	4,91E-01
	LTD 30	-	2,13	14	0,13							
	Control	+	1,14	14	0,09							
	LTD 30	+	1,04	14	0,16							

B)

Bonferroni adjustment: 95% confidence interval (*) p < 1.25E-02; 99% confidence interval (**) p < 2.50E-03; 99,9% confidence interval (***) p < 2.50E-04

H3K27Me3S28p

		SB-203580	Mean	N	SEM	Kruskal-Wallis Test	Mann-Whitney U Test Control/LTD10	Control/Control +	Control/LTD10+	LTD10/Control+	LTD10/LTD10+	Control+/LTD10+
Bdnf Promoter I	Control	-	1,00	7	0,00	5,06E-01	1,52E-01	3,26E-01	3,26E-01	4,25E-01	4,25E-01	8,27E-01
	LTD 10	-	1,34	7	0,30							
	Control	+	0,95	3	0,27							
	LTD 10	+	0,94	3	0,22							
Bdnf Promoter II	Control	-	1,00	6	0,00	1,93E-03	1,11E-03	3,26E-01	3,24E-03	2,01E-02	2,01E-02	5,13E-01
	LTD 10	-	2,10	6	0,53							
	Control	+	0,94	3	0,10							
	LTD 10	+	0,80	3	0,10							
Bdnf Promoter IV	Control	-	1,00	7	0,00	6,03E-01	6,33E-01	3,26E-01	3,70E-02	5,69E-01	9,09E-01	5,13E-01
	LTD 10	-	1,11	7	0,26							
	Control	+	1,25	3	0,39							
	LTD 10	+	1,29	3	0,09							
Bdnf Promoter VI	Control	-	1,00	6	0,00	2,63E-03	1,11E-03	3,24E-03	3,26E-01	7,07E-02	2,01E-02	5,13E-01
	LTD 10	-	1,77	6	0,26							
	Control	+	1,14	3	0,10							
	LTD 10	+	0,97	3	0,08							

IgG

		SB-203580	Mean	N	SEM
Bdnf Promoter I	Control	-	0,212	7	0,049
	LTD 10	-	0,165	7	0,058
	Control	+	0,238	3	0,086
	LTD 10	+	0,364	3	0,046
Bdnf Promoter II	Control	-	0,289	6	0,071
	LTD 10	-	0,206	6	0,070
	Control	+	0,278	3	0,071
	LTD 10	+	0,239	3	0,111
Bdnf Promoter IV	Control	-	0,284	7	0,040
	LTD 10	-	0,229	7	0,076
	Control	+	0,173	3	0,069
	LTD 10	+	0,166	3	0,095
Bdnf Promoter VI	Control	-	0,336	6	0,035
	LTD 10	-	0,254	6	0,054
	Control	+	0,122	3	0,014
	LTD 10	+	0,146	3	0,034

C)

Bonferroni adjustment: 95% confidence interval (*) p < 1.25E-02; 99% confidence interval (**) p < 2.50E-03; 99,9% confidence interval (***) p < 2.50E-04

EZH2

		SB-203580	Mean	N	SEM	Kruskal-Wallis Test	Mann-Whitney U Test Control/LTD10	Control/Control +	Control/LTD10+	LTD10/Control+	LTD10/LTD10+	Control+/LTD10+
Bdnf Promoter I	Control	-	1,00	5	0,00	2,66E-01	5,77E-01	1,80E-01	1,80E-01	4,62E-01	8,64E-02	2,48E-01
	LTD 10	-	0,91	5	0,13							
	Control	+	1,12	4	0,15							
	LTD 10	+	1,50	4	0,26							
Bdnf Promoter II	Control	-	1,00	6	0,00	1,68E-03	3,35E-03	1,87E-01	8,30E-03	1,05E-02	1,05E-02	5,64E-01
	LTD 10	-	0,54	6	0,12							
	Control	+	1,09	4	0,06							
	LTD 10	+	1,18	4	0,07							
Bdnf Promoter IV	Control	-	1,00	6	0,00	5,69E-03	2,09E-03	1,00E+00	1,00E+00	1,05E-02	1,05E-02	2,48E-01
	LTD 10	-	0,57	6	0,03							
	Control	+	1,05	4	0,12							
	LTD 10	+	1,37	4	0,27							
Bdnf Promoter VI	Control	-	1,00	6	0,00	1,68E-03	3,35E-03	1,87E-01	8,30E-03	1,05E-02	1,05E-02	5,64E-01
	LTD 10	-	0,54	6	0,12							
	Control	+	1,09	4	0,06							
	LTD 10	+	1,18	4	0,07							

IgG

		SB-203580	Mean	N	SEM
Bdnf Promoter I	Control	-	0,122	5	0,032
	LTD 10	-	0,126	5	0,053
	Control	+	0,048	4	0,007
	LTD 10	+	0,069	4	0,016
Bdnf Promoter II	Control	-	0,166	6	0,026
	LTD 10	-	0,123	6	0,050
	Control	+	0,063	4	0,021
	LTD 10	+	0,094	4	0,041
Bdnf Promoter IV	Control	-	0,157	6	0,033
	LTD 10	-	0,091	6	0,032
	Control	+	0,053	4	0,013
	LTD 10	+	0,103	4	0,047
Bdnf Promoter VI	Control	-	0,152	6	0,041
	LTD 10	-	0,066	6	0,017
	Control	+	0,069	4	0,010
	LTD 10	+	0,132	4	0,049

D)

Bonferroni adjustment	95% confidence interval	99% confidence interval	99,9% confidence interval
	(*) p < 1.25E-02	(**) p < 2.50E-03	(***) p < 2.50E-04

		GSK-J4	Mean	N	SEM	Kruskal-Wallis Test	Mann-Whitney U Test					
							Control/LTD30	Control/Control +	Control/LTD30+	LTD30/Control+	LTD30/LTD30+	Control+/LTD30+
Bdnf Transcript I	Control	-	1,00	13	0,00	3,75E-03	1,04E-02	1,87E-03	4,55E-04	2,51E-01	6,16E-01	2,66E-01
	LTD 30	-	1,34	13	0,13							
	Control	+	1,15	9	0,09							
	LTD 30	+	1,57	9	0,21							
Bdnf Transcript II	Control	-	1,00	13	0,00	9,55E-05	4,76E-06	5,46E-01	3,12E-02	9,80E-04	3,87E-04	4,18E-01
	LTD 30	-	2,07	12	0,15							
	Control	+	0,89	8	0,17							
	LTD 30	+	0,77	8	0,12							
Bdnf Transcript IV	Control	-	1,00	13	0,00	1,29E-02	3,64E-06	1,67E-01	6,52E-04	6,55E-02	4,43E-01	1,26E-01
	LTD 30	-	1,31	13	0,06							
	Control	+	1,12	9	0,10							
	LTD 30	+	1,52	9	0,18							
Bdnf Transcript VI	Control	-	1,00	14	0,00	3,37E-02	1,49E-06	5,34E-01	2,68E-01	3,62E-03	1,74E-04	3,55E-01
	LTD 30	-	2,13	14	0,13							
	Control	+	1,04	8	0,22							
	LTD 30	+	1,11	8	0,12							

E)

Bonferroni adjustment	95% confidence interval	99% confidence interval	99,9% confidence interval
	(*) p < 1.25E-02	(**) p < 2.50E-03	(***) p < 2.50E-04

H3K27Me3

		GSK-J4	Mean	N	SEM	Kruskal-Wallis Test	Mann-Whitney U Test					
							Control/LTD10	Control/Control +	Control/LTD10+	LTD10/Control+	LTD10/LTD10+	Control+/LTD10+
Bdnf Promoter I	Control	-	1,00	7	0,00	6,16E-01	1,52E-01	3,26E-01	3,26E-01	5,69E-01	5,69E-01	8,27E-01
	LTD 10	-	1,00	7	0,14							
	Control	+	1,14	3	0,27							
	LTD 10	+	1,16	3	0,20							
Bdnf Promoter II	Control	-	1,00	7	0,00	9,60E-01	6,33E-01	3,26E-01	3,26E-01	9,09E-01	9,09E-01	5,13E-01
	LTD 10	-	1,15	7	0,16							
	Control	+	1,21	3	0,27							
	LTD 10	+	1,05	3	0,18							
Bdnf Promoter IV	Control	-	1,00	6	0,00	2,11E-02	6,01E-04	2,99E-01	2,99E-01	1,97E-01	7,07E-02	5,13E-01
	LTD 10	-	0,87	6	0,05							
	Control	+	1,06	3	0,15							
	LTD 10	+	1,18	3	0,14							
Bdnf Promoter VI	Control	-	1,00	6	0,00	5,06E-03	1,11E-03	3,26E-01	3,26E-01	2,01E-02	2,01E-02	8,27E-01
	LTD 10	-	0,59	6	0,04							
	Control	+	0,99	3	0,12							
	LTD 10	+	1,01	3	0,03							

IgG

		GSK-J4	Mean	N	SEM
Bdnf Promoter I	Control	-	0,003	7	0,001
	LTD 10	-	0,007	7	0,003
	Control	+	0,009	3	0,005
	LTD 10	+	0,009	3	0,005
Bdnf Promoter II	Control	-	0,006	7	0,002
	LTD 10	-	0,014	7	0,010
	Control	+	0,008	3	0,006
	LTD 10	+	0,008	3	0,006
Bdnf Promoter IV	Control	-	0,007	6	0,002
	LTD 10	-	0,008	6	0,004
	Control	+	0,009	3	0,003
	LTD 10	+	0,009	3	0,003
Bdnf Promoter VI	Control	-	0,005	6	0,002
	LTD 10	-	0,006	6	0,004
	Control	+	0,007	3	0,003
	LTD 10	+	0,007	3	0,003

F)

Bonferroni adjustment	95% confidence interval	99% confidence interval	99,9% confidence interval
	(*) p < 1.25E-02	(**) p < 2.50E-03	(***) p < 2.50E-04

		CCIIh	Mean	N	SEM	Kruskal-Wallis Test	Mann-Whitney U Test					
							Control/LTD30	Control/Control +	Control/LTD30+	LTD30/Control+	LTD30/LTD30+	Control+/LTD30+
Bdnf Transcript I	Control	-	1,00	13	0,00	4,64E-04	1,04E-02	5,34E-01	9,25E-06	1,91E-01	8,22E-02	7,77E-03
	LTD 30	-	1,34	13	0,13							
	Control	+	1,13	8	0,10							
	LTD 30	+	1,69	8	0,13							
Bdnf Transcript II	Control	-	1,00	13	0,00	4,08E-04	4,76E-06	5,46E-01	1,00E+00	3,86E-04	2,62E-03	8,17E-01
	LTD 30	-	2,07	12	0,15							
	Control	+	0,92	8	0,07							
	LTD 30	+	1,01	8	0,20							
Bdnf Transcript IV	Control	-	1,00	13	0,00	8,58E-05	3,64E-06	3,39E-05	1,64E-05	1,14E-01	5,97E-02	9,82E-03
	LTD 30	-	1,31	13	0,06							
	Control	+	1,18	8	0,08							
	LTD 30	+	1,57	8	0,08							
Bdnf Transcript VI	Control	-	1,00	14	0,00	1,87E-03	1,49E-06	5,34E-01	1,95E-05	2,57E-04	1,30E-03	6,64E-03
	LTD 30	-	2,13	14	0,13							
	Control	+	1,07	7	0,11							
	LTD 30	+	1,49	7	0,04							

G)

Bonferroni adjustment: 95% confidence interval (*) p < 1.25E-02; 99% confidence interval (**) p < 2.50E-03; 99,9% confidence interval (***) p < 2.50E-04

H3K27Ac			CCIIh	Mean	N	SEM	Kruskal-Wallis Test	Mann-Whitney U Test Control/LTD10	Control/Control +	Control/LTD10+	LTD10/Control+	LTD10/LTD10+	Control+/LTD10+
	Bdnf Promoter I	Control	-	1,00	7	0,00	9,92E-01	6,33E-01	5,26E-01	5,26E-01	8,08E-01	9,35E-01	9,17E-01
		LTD 10	-	0,91	7	0,14							
		Control	+	0,89	5	0,20							
		LTD 10	+	0,86	5	0,21							
	Bdnf Promoter II	Control	-	1,00	6	0,00	3,83E-03	1,53E-03	5,26E-01	5,72E-02	9,02E-03	9,02E-03	2,51E-01
		LTD 10	-	1,36	6	0,06							
		Control	+	0,88	5	0,14							
		LTD 10	+	0,70	5	0,13							
	Bdnf Promoter IV	Control	-	1,00	7	0,00	3,99E-01	1,19E-02	5,26E-01	5,26E-01	9,35E-01	6,85E-01	9,17E-01
		LTD 10	-	0,86	7	0,07							
		Control	+	0,98	5	0,35							
		LTD 10	+	0,98	5	0,24							
	Bdnf Promoter VI	Control	-	1,00	6	0,00	8,90E-04	2,09E-03	7,32E-02	2,82E-03	6,17E-03	6,17E-03	7,54E-01
		LTD 10	-	1,50	6	0,18							
		Control	+	0,72	5	0,13							
		LTD 10	+	0,79	5	0,09							

IgG			CCIIh	Mean	N	SEM
	Bdnf Promoter I	Control	-	0,031	7	0,006
		LTD 10	-	0,030	7	0,010
		Control	+	0,091	5	0,012
		LTD 10	+	0,082	5	0,036
	Bdnf Promoter II	Control	-	0,065	6	0,015
		LTD 10	-	0,070	6	0,033
		Control	+	0,072	5	0,016
		LTD 10	+	0,137	5	0,047
	Bdnf Promoter IV	Control	-	0,050	7	0,011
		LTD 10	-	0,040	7	0,014
		Control	+	0,106	5	0,024
		LTD 10	+	0,068	5	0,033
	Bdnf Promoter VI	Control	-	0,027	6	0,005
		LTD 10	-	0,036	6	0,010
		Control	+	0,056	5	0,015
		LTD 10	+	0,145	5	0,078

H)

Bonferroni adjustment: 95% confidence interval (*) p < 1.25E-02; 99% confidence interval (**) p < 2.50E-03; 99,9% confidence interval (***) p < 2.50E-04

CREB			CCIIh	Mean	N	SEM	Kruskal-Wallis Test	Mann-Whitney U Test Control/LTD10	Control/Control +	Control/LTD10+	LTD10/Control+	LTD10/LTD10+	Control+/LTD10+
	Bdnf Promoter I	Control	-	1,00	5	0,00	3,42E-03	5,35E-03	1,04E-02	5,35E-03	2,53E-02	6,02E-01	5,26E-02
		LTD 10	-	2,50	5	0,44							
		Control	+	1,19	5	0,09							
		LTD 10	+	2,12	5	0,29							
	Bdnf Promoter II	Control	-	1,00	5	0,00	2,22E-03	5,35E-03	1,04E-02	5,35E-03	2,53E-02	1,75E-01	2,53E-02
		LTD 10	-	2,23	5	0,40							
		Control	+	0,84	5	0,11							
		LTD 10	+	2,45	5	0,10							
	Bdnf Promoter IV	Control	-	1,00	5	0,00	6,34E-03	5,35E-03	3,93E-01	5,35E-03	2,53E-02	9,17E-01	5,26E-02
		LTD 10	-	2,74	5	0,84							
		Control	+	1,04	5	0,12							
		LTD 10	+	2,94	5	0,83							
	Bdnf Promoter VI	Control	-	1,00	5	0,00	4,59E-03	5,35E-03	3,93E-01	5,35E-03	2,53E-02	9,17E-01	2,53E-02
		LTD 10	-	2,42	5	0,30							
		Control	+	0,98	5	0,10							
		LTD 10	+	2,93	5	0,80							

IgG			CCIIh	Mean	N	SEM
	Bdnf Promoter I	Control	-	0,346	5	0,020
		LTD 10	-	0,302	5	0,015
		Control	+	0,300	5	0,122
		LTD 10	+	0,401	5	0,071
	Bdnf Promoter II	Control	-	0,506	5	0,113
		LTD 10	-	0,500	5	0,057
		Control	+	0,448	5	0,106
		LTD 10	+	0,668	5	0,089
	Bdnf Promoter IV	Control	-	0,545	5	0,085
		LTD 10	-	0,479	5	0,133
		Control	+	0,430	5	0,067
		LTD 10	+	0,506	5	0,067
	Bdnf Promoter VI	Control	-	0,593	5	0,115
		LTD 10	-	0,646	5	0,073
		Control	+	0,430	5	0,150
		LTD 10	+	0,654	5	0,041

I)

Bonferroni adjustment: 95% confidence interval (*) p < 1.25E-02; 99% confidence interval (**) p < 2.50E-03; 99,9% confidence interval (***) p < 2.50E-04

CBP			CCIIh	Mean	N	SEM	Kruskal-Wallis Test	Mann-Whitney U Test Control/LTD10	Control/Control +	Control/LTD10+	LTD10/Control+	LTD10/LTD10+	Control+/LTD10+
	Bdnf Promoter I	Control	-	1,00	5	0,00	8,04E-03	5,35E-03	3,93E-01	9,47E-02	5,26E-02	9,02E-03	1,80E-01
		LTD 10	-	1,77	5	0,10							
		Control	+	1,16	5	0,16							
		LTD 10	+	0,77	5	0,14							
	Bdnf Promoter II	Control	-	1,00	5	0,00	1,03E-02	5,35E-03	3,93E-01	9,47E-02	5,26E-02	9,02E-03	4,56E-01
		LTD 10	-	1,87	5	0,27							
		Control	+	0,92	5	0,19							
		LTD 10	+	0,78	5	0,12							
	Bdnf Promoter IV	Control	-	1,00	5	0,00	8,48E-03	5,35E-03	3,93E-01	5,77E-01	2,53E-02	9,02E-03	1,01E-01
		LTD 10	-	2,07	5	0,17							
		Control	+	0,81	5	0,16							
		LTD 10	+	1,12	5	0,15							
	Bdnf Promoter VI	Control	-	1,00	5	0,00	1,29E-02	5,35E-03	3,93E-01	5,77E-01	2,53E-02	9,02E-03	6,55E-01
		LTD 10	-	2,81	5	0,28							
		Control	+	0,98	5	0,07							
		LTD 10	+	1,08	5	0,16							

IgG			CCIIh	Mean	N	SEM
	Bdnf Promoter I	Control	-	0,105	5	0,026
		LTD 10	-	0,125	5	0,045
		Control	+	0,037	5	0,017
		LTD 10	+	0,207	5	0,059
	Bdnf Promoter II	Control	-	0,116	5	0,034
		LTD 10	-	0,118	5	0,037
		Control	+	0,074	5	0,057
		LTD 10	+	0,160	5	0,053
	Bdnf Promoter IV	Control	-	0,181	5	0,061
		LTD 10	-	0,073	5	0,003
		Control	+	0,058	5	0,022
		LTD 10	+	0,180	5	0,121
	Bdnf Promoter VI	Control	-	0,122	5	0,047
		LTD 10	-	0,127	5	0,049
		Control	+	0,047	5	0,019
		LTD 10	+	0,133	5	0,070

J)

Bonferroni adjustment: 95% confidence interval (*) p < 1.25E-02; 99% confidence interval (**) p < 2.50E-03; 99,9% confidence interval (***) p < 2.50E-04

JMJD3		CCIIh	Mean	N	SEM	Kruskal-Wallis Test	Mann-Whitney U Test Control/LTD10	Control/Control +	Control/LTD10+	LTD10/Control+	LTD10/LTD10+	Control+/LTD10+
Bdnf Promoter I	Control	-	1,00	4	0,00	8,29E-01	2,19E-01	4,35E-01	4,35E-01	1,00E+00	4,80E-01	5,13E-01
	LTD 10	-	1,23	4	0,26							
	Control	+	1,11	3	0,38							
	LTD 10	+	1,00	3	0,09							
Bdnf Promoter II	Control	-	1,00	4	0,00	8,50E-03	1,19E-02	1,93E-02	1,93E-02	3,39E-02	3,39E-02	8,27E-01
	LTD 10	-	1,47	4	0,06							
	Control	+	0,75	3	0,09							
	LTD 10	+	0,78	3	0,11							
Bdnf Promoter IV	Control	-	1,00	4	0,00	2,22E-02	1,19E-02	4,35E-01	1,93E-02	7,71E-02	3,39E-02	2,75E-01
	LTD 10	-	1,80	4	0,24							
	Control	+	1,02	3	0,35							
	LTD 10	+	0,59	3	0,17							
Bdnf Promoter VI	Control	-	1,00	4	0,00	2,77E-02	1,19E-02	4,35E-01	2,19E-01	3,39E-02	2,09E-02	7,24E-01
	LTD 10	-	1,55	4	0,10							
	Control	+	0,76	3	0,20							
	LTD 10	+	0,84	4	0,14							

IgG		CCIIh	Mean	N	SEM
Bdnf Promoter I	Control	-	0,084	4	0,013
	LTD 10	-	0,083	4	0,012
	Control	+	0,069	3	0,029
	LTD 10	+	0,056	3	0,028
Bdnf Promoter II	Control	-	0,073	4	0,011
	LTD 10	-	0,091	4	0,023
	Control	+	0,019	3	0,015
	LTD 10	+	0,139	3	0,084
Bdnf Promoter IV	Control	-	0,138	4	0,039
	LTD 10	-	0,107	4	0,023
	Control	+	0,224	3	0,109
	LTD 10	+	0,082	3	0,053
Bdnf Promoter VI	Control	-	0,102	4	0,027
	LTD 10	-	0,083	4	0,011
	Control	+	0,089	3	0,027
	LTD 10	+	0,053	4	0,025

K)

Bonferroni adjustment: 95% confidence interval (*) p < 1.25E-02; 99% confidence interval (**) p < 2.50E-03; 99,9% confidence interval (***) p < 2.50E-04

H3K27Me3		CCIIh	Mean	N	SEM	Kruskal-Wallis Test	Mann-Whitney U Test Control/LTD10	Control/Control +	Control/LTD10+	LTD10/Control+	LTD10/LTD10+	Control+/LTD10+
Bdnf Promoter I	Control	-	1,00	7	0,00	5,32E-01	1,52E-01	3,26E-01	3,26E-01	2,10E-01	7,32E-01	5,13E-01
	LTD 10	-	1,00	7	0,14							
	Control	+	1,01	3	0,07							
	LTD 10	+	1,23	3	0,31							
Bdnf Promoter II	Control	-	1,00	7	0,00	2,33E-01	6,33E-01	3,26E-01	3,24E-03	5,69E-01	2,10E-01	5,13E-01
	LTD 10	-	1,15	7	0,16							
	Control	+	1,18	3	0,17							
	LTD 10	+	1,51	3	0,28							
Bdnf Promoter IV	Control	-	1,00	6	0,00	5,14E-03	6,01E-04	1,84E-03	2,99E-01	2,01E-02	3,02E-01	5,13E-01
	LTD 10	-	0,87	6	0,05							
	Control	+	1,44	3	0,32							
	LTD 10	+	1,24	3	0,33							
Bdnf Promoter VI	Control	-	1,00	6	0,00	5,04E-03	1,11E-03	3,57E-01	3,57E-01	2,01E-02	2,01E-02	1,27E-01
	LTD 10	-	0,59	6	0,04							
	Control	+	0,94	3	0,03							
	LTD 10	+	1,35	3	0,18							

IgG		CCIIh	Mean	N	SEM
Bdnf Promoter I	Control	-	0,003	7	0,001
	LTD 10	-	0,007	7	0,003
	Control	+	0,006	3	0,001
	LTD 10	+	0,006	3	0,001
Bdnf Promoter II	Control	-	0,006	7	0,002
	LTD 10	-	0,014	7	0,010
	Control	+	0,005	3	0,002
	LTD 10	+	0,005	3	0,002
Bdnf Promoter IV	Control	-	0,007	6	0,002
	LTD 10	-	0,008	6	0,004
	Control	+	0,007	3	0,001
	LTD 10	+	0,007	3	0,001
Bdnf Promoter VI	Control	-	0,005	6	0,002
	LTD 10	-	0,006	6	0,004
	Control	+	0,006	3	0,001
	LTD 10	+	0,006	3	0,001

Supplementary Table 4: Data and statistics corresponding to Figure 4

A)

Bonferroni adjustment	95% confidence interval	99% confidence interval	99.9% confidence interval
	(*) p < 1.67E-02	(**) p < 3.33E-03	(***) p < 3.33E-04

	min	Mean	N	SEM	Kruskal-Wallis Test	Mann-Whitney U Test		
						Control / LTD 10	Control / LTD 30	LTD10 / LTD 30
Bdnf Transctipt I	0	1.00	8	0.00				
	10	0.98	6	0.08	6.73E-03	1.67E-02	7.10E-03	3.89E-02
	30	1.24	8	0.09				

	min	Mean	N	SEM	Kruskal-Wallis Test	Mann-Whitney U Test		
						Control / LTD 10	Control / LTD 30	LTD10 / LTD 30
Bdnf Transctipt II	0	1.00	8	0.00				
	10	1.30	6	0.11	1.09E-04	3.31E-04	2.55E-04	9.90E-02
	30	1.52	8	0.10				

	min	Mean	N	SEM	Kruskal-Wallis Test	Mann-Whitney U Test		
						Control / LTD 10	Control / LTD 30	LTD10 / LTD 30
Bdnf Transctipt VI	0	1.00	8	0.00				
	10	1.00	6	0.12	1.05E-02	1.00E+00	4.40E-04	1.99E-01
	30	1.25	7	0.06				

	min	Mean	N	SEM	Kruskal-Wallis Test	Mann-Whitney U Test		
						Control / LTD 10	Control / LTD 30	LTD10 / LTD 30
Bdnf Transctipt VI	0	1.00	8	0.00				
	10	1.28	5	0.09	1.69E-04	4.72E-04	3.31E-04	1.07E-01
	30	1.49	8	0.10				

B)

		Mean	N	SEM
H3K27Me3	*Bdnf* Promoter I	2,62	4	0,63
	Bdnf Promoter II	2,08	4	0,21
	Bdnf Promoter IV	2,09	4	0,48
	Bdnf Promoter VI	2,67	4	0,11
	ßActin	0,37	4	0,12
	hoxA1	6,43	4	1,15

		Mean	N	SEM
IgG	*Bdnf* Promoter I	0,027	4	0,010
	Bdnf Promoter II	0,042	4	0,018
	Bdnf Promoter IV	0,028	4	0,013
	Bdnf Promoter VI	0,044	4	0,008
	ßActin	0,029	4	0,008
	hoxA1	0,021	4	0,007

C)

		Mean	N	SEM
EZH2	*Bdnf* Promoter I	2,73	6	0,49
	Bdnf Promoter II	2,90	6	0,53
	Bdnf Promoter IV	2,64	6	0,46
	Bdnf Promoter VI	3,50	6	0,65
	ßActin	1,25	6	0,23
	hoxA1	5,46	6	0,53

		Mean	N	SEM
IgG	*Bdnf* Promoter I	0,034	6	0,012
	Bdnf Promoter II	0,029	6	0,008
	Bdnf Promoter IV	0,022	6	0,012
	Bdnf Promoter VI	0,034	6	0,009
	ßActin	0,025	6	0,007
	hoxA1	0,027	6	0,009

D)

		Mean	N	SEM
JMJD3	*Bdnf* Promoter I	0,47	5	0,07
	Bdnf Promoter II	0,45	5	0,06
	Bdnf Promoter IV	0,52	5	0,07
	Bdnf Promoter VI	0,59	5	0,12
	ßActin	0,40	5	0,09
	hoxA1	0,08	5	0,02

		Mean	N	SEM
IgG	*Bdnf* Promoter I	0,053	5	0,033
	Bdnf Promoter II	0,040	5	0,014
	Bdnf Promoter IV	0,079	5	0,034
	Bdnf Promoter VI	0,044	5	0,019
	ßActin	0,066	5	0,016
	hoxA1	0,070	5	0,029

E)

		Mean	N	SEM
H3K4Me3	*Bdnf* Promoter I	5,36	4	0,76
	Bdnf Promoter II	1,50	4	0,09
	Bdnf Promoter IV	1,21	4	0,17
	Bdnf Promoter VI	5,74	4	0,65
	ßActin	6,25	4	0,46
	hoxA1	2,29	4	0,22

		Mean	N	SEM
IgG	*Bdnf* Promoter I	0,027	4	0,010
	Bdnf Promoter II	0,042	4	0,018
	Bdnf Promoter IV	0,028	4	0,013
	Bdnf Promoter VI	0,044	4	0,008
	ßActin	0,029	4	0,008
	hoxA1	0,021	4	0,007

F)

		Mean	N	SEM
H3K27Ac	*Bdnf* Promoter I	0,40	4	0,05
	Bdnf Promoter II	0,11	4	0,03
	Bdnf Promoter IV	0,73	4	0,14
	Bdnf Promoter VI	0,49	4	0,06
	ßActin	1,15	4	0,16
	hoxA1	0,08	4	0,03

		Mean	N	SEM
IgG	*Bdnf* Promoter I	0,027	4	0,010
	Bdnf Promoter II	0,042	4	0,018
	Bdnf Promoter IV	0,028	4	0,013
	Bdnf Promoter VI	0,044	4	0,008
	ßActin	0,029	4	0,008
	hoxA1	0,021	4	0,007

G)

		Mean	N	SEM	Mann-Whitney U Test
Bdnf Promoter I	EZH2	0,79	6	0,05	2,09E-03
	JMJD3	2,19	6	0,32	2,09E-03
	H3K4Me3	2,19	6	0,32	1,69E-02
	H3K27Ac	1,24	7	0,31	1,00E+00
	H3K27Me3	0,97	6	0,19	1,52E-01
	H3K27Me3S28p	1,46	7	0,13	7,10E-03

			Mean	N	SEM
Bdnf Promoter I	EZH2	IgG	0,012	6	0,0048
	JMJD3	IgG	0,072	6	0,0218
	H3K4Me3	IgG	0,061	6	0,0244
	H3K27Ac	IgG	0,019	7	0,0047
	H3K27Me3	IgG	0,001	6	0,0005
	H3K27Me3S28p	IgG	0,075	7	0,0202

		Mean	N	SEM	Mann-Whitney U Test
Bdnf Promoter II	EZH2	0,81	6	0,04	2,09E-03
	JMJD3	2,42	6	0,41	2,09E-03
	H3K4Me3	1,32	6	0,10	2,09E-03
	H3K27Ac	1,60	6	0,29	4,03E-02
	H3K27Me3	0,80	7	0,11	1,69E-02
	H3K27Me3S28p	1,59	7	0,33	1,69E-02

			Mean	N	SEM
Bdnf Promoter II	EZH2	IgG	0,029	6	0,0170
	JMJD3	IgG	0,061	6	0,0244
	H3K4Me3	IgG	0,001	6	0,0004
	H3K27Ac	IgG	0,068	6	0,0345
	H3K27Me3	IgG	0,001	7	0,0004
	H3K27Me3S28p	IgG	0,064	7	0,0385

		Mean	N	SEM	Mann-Whitney U Test
Bdnf Promoter IV	EZH2	0,74	6	0,06	2,09E-03
	JMJD3	2,14	6	0,30	2,09E-03
	H3K4Me3	1,32	6	0,10	2,09E-03
	H3K27Ac	1,05	6	0,25	3,05E-01
	H3K27Me3	0,70	6	0,10	2,97E-02
	H3K27Me3S28p	1,34	6	0,15	4,03E-02

			Mean	N	SEM
Bdnf Promoter IV	EZH2	IgG	0,012	6	0,0041
	JMJD3	IgG	0,158	6	0,0644
	H3K4Me3	IgG	0,001	6	0,0004
	H3K27Ac	IgG	0,029	6	0,0188
	H3K27Me3	IgG	0,001	6	0,0005
	H3K27Me3S28p	IgG	0,070	6	0,0393

		Mean	N	SEM	Mann-Whitney U Test
Bdnf Promoter VI	EZH2	0,75	6	0,05	2,09E-03
	JMJD3	2,42	6	0,49	2,09E-03
	H3K4Me3	1,36	6	0,08	2,09E-03
	H3K27Ac	1,54	7	0,24	1,69E-02
	H3K27Me3	0,68	7	0,09	1,69E-02
	H3K27Me3S28p	1,34	7	0,11	1,69E-02

			Mean	N	SEM
Bdnf Promoter VI	EZH2	IgG	0,018	6	0,0099
	JMJD3	IgG	0,057	6	0,0249
	H3K4Me3	IgG	0,000	6	0,0002
	H3K27Ac	IgG	0,013	7	0,0066
	H3K27Me3	IgG	0,001	7	0,0003
	H3K27Me3S28p	IgG	0,034	7	0,0185

H)

		Mean	N	SEM	Mann-Whitney U Test
ßActin	EZH2	0,95	6	0,11	3,05E-01
	JMJD3	1,73	6	0,21	2,82E-03
	H3K4Me3	0,91	6	0,22	6,33E-01
	H3K27Ac	1,20	7	0,39	7,27E-02
	H3K27Me3	0,88	7	0,19	6,33E-01
	H3K27Me3S28p	1,33	7	0,33	6,33E-01

			Mean	N	SEM
ßActin	EZH2	IgG	0,018	6	0,0089
	JMJD3	IgG	0,109	6	0,0639
	H3K4Me3	IgG	0,091	6	0,0330
	H3K27Ac	IgG	0,001	7	0,0004
	H3K27Me3	IgG	0,145	7	0,1397
	H3K27Me3S28p	IgG	0,401	7	0,2598

		Mean	N	SEM	Mann-Whitney U Test
hoxA1	EZH2	0,79	6	0,13	9,47E-02
	JMJD3	1,32	6	0,17	9,47E-02
	H3K4Me3	0,96	6	0,21	6,33E-01
	H3K27Ac	1,27	7	0,35	6,33E-01
	H3K27Me3	0,92	7	0,10	1,52E-01
	H3K27Me3S28p	1,09	7	0,06	2,77E-01

			Mean	N	SEM
hoxA1	EZH2	IgG	0,028	6	0,0170
	JMJD3	IgG	0,091	6	0,0330
	H3K4Me3	IgG	0,001	6	0,0005
	H3K27Ac	IgG	0,099	7	0,0237
	H3K27Me3	IgG	0,002	7	0,0008
	H3K27Me3S28p	IgG	0,137	7	0,1210

Supplementary Table 5: Data and statistics corresponding to Supplementary Figure 1

A)

		Mean	N	SEM	Mann-Whitney U Test
Bdnf Promoter I	EZH2	1,20	5	0,21	5,77E-01
	JMJD3	1,27	4	0,31	2,19E-01
	H3K4Me3	0,92	6	0,16	2,77E-01
	H3K27Ac	0,61	5	0,17	5,72E-02
	H3K27Me3	0,96	5	0,22	5,26E-01
	H3K27Me3S28p	1,13	6	0,21	1,00E+00
Bdnf Promoter II	EZH2	1,06	5	0,14	9,26E-02
	JMJD3	0,95	4	0,13	1,00E+00
	H3K4Me3	0,67	6	0,14	4,03E-02
	H3K27Ac	0,54	5	0,12	1,53E-03
	H3K27Me3	1,01	5	0,20	5,26E-01
	H3K27Me3S28p	0,71	6	0,17	2,77E-01
Bdnf Promoter IV	EZH2	1,41	5	0,22	5,50E-01
	JMJD3	1,09	4	0,23	1,00E+00
	H3K4Me3	0,86	6	0,17	2,77E-01
	H3K27Ac	0,49	5	0,16	1,53E-03
	H3K27Me3	0,77	5	0,11	4,52E-02
	H3K27Me3S28p	1,02	5	0,37	1,25E-01
Bdnf Promoter VI	EZH2	1,06	5	0,14	9,26E-02
	JMJD3	0,99	4	0,17	1,00E+00
	H3K4Me3	0,92	6	0,17	1,00E+00
	H3K27Ac	0,54	5	0,10	2,82E-03
	H3K27Me3	1,00	6	0,20	5,26E-01
	H3K27Me3S28p	1,06	6	0,21	2,77E-01

			Mean	N	SEM
Bdnf Promoter I	EZH2	IgG	0,101	5	0,023
	JMJD3	IgG	0,069	4	0,029
	H3K4Me3	IgG	0,007	6	0,003
	H3K27Ac	IgG	0,031	5	0,008
	H3K27Me3	IgG	0,005	5	0,002
	H3K27Me3S28p	IgG	0,285	6	0,108
Bdnf Promoter II	EZH2	IgG	0,110	5	0,026
	JMJD3	IgG	0,068	4	0,025
	H3K4Me3	IgG	0,008	6	0,003
	H3K27Ac	IgG	0,031	5	0,008
	H3K27Me3	IgG	0,002	5	0,001
	H3K27Me3S28p	IgG	0,175	6	0,123
Bdnf Promoter IV	EZH2	IgG	0,108	5	0,035
	JMJD3	IgG	0,161	4	0,075
	H3K4Me3	IgG	0,008	6	0,003
	H3K27Ac	IgG	0,024	5	0,010
	H3K27Me3	IgG	0,004	5	0,001
	H3K27Me3S28p	IgG	0,179	5	0,056
Bdnf Promoter VI	EZH2	IgG	0,119	5	0,026
	JMJD3	IgG	0,235	4	0,063
	H3K4Me3	IgG	0,006	6	0,003
	H3K27Ac	IgG	0,018	5	0,004
	H3K27Me3	IgG	0,003	6	0,002
	H3K27Me3S28p	IgG	0,351	6	0,106

B)

		Mean	N	SEM	Mann-Whitney U Test
ßActin	EZH2	1,45	5	0,30	9,47E-02
	JMJD3	1,27	4	0,08	1,39E-02
	H3K4Me3	1,10	6	0,13	2,77E-01
	H3K27Ac	0,68	5	0,07	1,53E-03
	H3K27Me3	0,86	5	0,15	5,26E-01
	H3K27Me3S28p	1,27	5	0,37	5,26E-01
hoxA1	EZH2	1,14	5	0,36	5,50E-01
	JMJD3	1,12	4	0,21	1,00E+00
	H3K4Me3	0,83	6	0,08	5,72E-02
	H3K27Ac	0,69	5	0,10	1,53E-03
	H3K27Me3	0,87	5	0,17	5,26E-01
	H3K27Me3S28p	0,92	6	0,10	2,77E-01

			Mean	N	SEM
ßActin	EZH2	IgG	0,210	5	0,047
	JMJD3	IgG	0,115	4	0,035
	H3K4Me3	IgG	0,013	6	0,007
	H3K27Ac	IgG	0,014	5	0,004
	H3K27Me3	IgG	0,044	5	0,009
	H3K27Me3S28p	IgG	0,310	5	0,120
hoxA1	EZH2	IgG	0,087	5	0,035
	JMJD3	IgG	0,107	4	0,019
	H3K4Me3	IgG	0,003	6	0,001
	H3K27Ac	IgG	0,153	5	0,054
	H3K27Me3	IgG	0,002	5	0,001
	H3K27Me3S28p	IgG	0,235	6	0,055

Supplementary Table 6: Data and statistics corresponding to Supplementary Figure 2

B)

		Mean	N	SEM
Cox2	MFEs p38 WT	0.042	3	0.001
	MFEs p38 KO	0.020	3	0.003

		Mean	N	SEM
IgG	MFEs p38 WT	0.020	3	0.006
	MFEs p38 KO	0.023	3	0.001

C)

		Mean	N	SEM	Mann-Whitney U Test
p38	*Bdnf* Promoter I	0.96	4	0.08	1.00E+00
	Bdnf Promoter II	2.04	4	0.22	1.39E-02
	Bdnf Promoter IV	0.93	4	0.13	1.00E+00
	Bdnf Promoter VI	1.91	4	0.16	1.39E-02

		Mean	N	SEM
IgG	*Bdnf* Promoter I	0.59	4	0.10
	Bdnf Promoter II	0.79	4	0.20
	Bdnf Promoter IV	0.99	4	0.29
	Bdnf Promoter VI	0.54	4	0.08

D)

		Mean	N	SEM	Mann-Whitney U Test
p38	*ßActin*	1.00	4	0.24	1.00E+00
	hoxA1	0.92	4	0.11	1.00E+00

		Mean	N	SEM
IgG	*ßActin*	1.11	4	0.21
	hoxA1	0.93	4	0.19

Supplementary Table 7: Data and statistics corresponding to Supplementary Figure 3

A)

Bonferroni adjustment: 95% confidence interval (*) p < 1.67E-02; 99% confidence interval (**) p < 3.33E-03; 99.9% confidence interval (***) p < 3.33E-04

		GSK-J4		Mean	N	SEM	Kruskal-Wallis Test	Mann-Whitney U Test Control/LTD10	Control/Control +	Control/LTD10+	LTD10/Control+	LTD10/LTD10+	Control+/LTD10+
H3K9Me3	*Bdnf* Promoter I	-	Control	1.00	4	0.00	9.84E-01	1.00E+00	1.00E+00	1.00E+00	5.64E-01	7.73E-01	7.73E-01
		-	LTD 10	1.00	4	0.05							
		+	Control	0.93	4	0.09							
		+	LTD 10	0.93	4	0.11							
	Bdnf Promoter II	-	Control	1.00	4	0.00	4.62E-01	2.19E-01	2.19E-01	2.19E-01	5.64E-01	7.73E-01	3.86E-01
		-	LTD 10	1.09	4	0.07							
		+	Control	1.17	4	0.13							
		+	LTD 10	1.08	4	0.11							
	Bdnf Promoter IV	-	Control	1.00	4	0.00	4.13E-01	2.19E-01	1.00E+00	2.19E-01	1.49E-01	7.73E-01	3.86E-01
		-	LTD 10	0.87	4	0.11							
		+	Control	1.11	4	0.15							
		+	LTD 10	0.97	4	0.27							
	Bdnf Promoter VI	-	Control	1.00	4	0.00	5.38E-01	2.19E-01	2.19E-01	2.19E-01	1.00E+00	7.73E-01	1.00E+00
		-	LTD 10	1.25	4	0.18							
		+	Control	1.24	4	0.23							
		+	LTD 10	1.22	4	0.14							

			GSK-J4	Mean	N	SEM
IgG	*Bdnf* Promoter I	Control	-	0.03	4	0.02
		LTD 10	-	0.06	4	0.05
		Control	+	0.03	4	0.02
		LTD 10	+	0.03	4	0.02
	Bdnf Promoter II	Control	-	0.02	4	0.01
		LTD 10	-	0.05	4	0.04
		Control	+	0.03	4	0.01
		LTD 10	+	0.05	4	0.02
	Bdnf Promoter IV	Control	-	0.02	4	0.01
		LTD 10	-	0.04	4	0.03
		Control	+	0.03	4	0.02
		LTD 10	+	0.06	4	0.03
	Bdnf Promoter VI	Control	-	0.02	4	0.01
		LTD 10	-	0.05	4	0.03
		Control	+	0.04	4	0.02
		LTD 10	+	0.08	4	0.04

B)

	Mean	N	SEM	Mann-Whitney U Test
Jmjd3	1.00	5	0.00	5.35E-03
	0.51	5	0.08	

C)

	Mean	N	SEM	Mann-Whitney U Test
JMJD3	0.00	4	0.00	2.09E-02
	0.12	4	0.06	

D)

		Lentivirus	Mean	N	SEM
JMJD3	*Bdnf* Promoter I	Scr	0.48	3	0.03
		Sh	0.06	3	0.04
	Bdnf Promoter II	Scr	0.75	3	0.05
		Sh	0.14	3	0.03
	Bdnf Promoter IV	Scr	0.81	3	0.05
		Sh	0.09	3	0.09
	Bdnf Promoter VI	Scr	0.85	3	0.19
		Sh	0.09	3	0.04

		Lentivirus	Mean	N	SEM
IgG	*Bdnf* Promoter I	Scr	0.04	3	0.03
		Sh	0.06	3	0.02
	Bdnf Promoter II	Scr	0.07	3	0.03
		Sh	0.09	3	0.01
	Bdnf Promoter IV	Scr	0.06	3	0.05
		Sh	0.01	3	0.01
	Bdnf Promoter VI	Scr	0.04	3	0.04
		Sh	0.05	3	0.01

E)

	Lentivirus		Mean	N	SEM	Mann-Whitney U Test Control/LTD10
Bdnf Promoter I	Sc	Control	1.00	3	0.00	3.69E-02
		LTD 30	1.41	3	0.09	
	Sh	Control	1.00	3	0.00	3.69E-02
		LTD 30	1.50	3	0.12	
Bdnf Promoter II	Sc	Control	1.00	3	0.00	3.69E-02
		LTD 30	1.57	3	0.18	
	Sh	Control	1.00	3	0.00	4.87E-01
		LTD 30	1.04	3	0.08	
Bdnf Promoter IV	Sc	Control	1.00	3	0.00	3.69E-02
		LTD 30	1.51	3	0.05	
	Sh	Control	1.00	3	0.00	3.69E-02
		LTD 30	1.29	3	0.01	
Bdnf Promoter VI	Sc	Control	1.00	3	0.00	3.69E-02
		LTD 30	1.35	3	0.02	
	Sh	Control	1.00	3	0.00	4.87E-01
		LTD 30	0.99	3	0.04	

F)

		Lentivirus		Mean	N	SEM	Mann-Whitney U Test Control/LTD10
H3K27Me3	*Bdnf* Promoter I	Sc	Control	1.00	5	0.00	5.77E-01
			LTD 10	0.99	5	0.18	
		Sh	Control	1.00	5	0.00	5.77E-01
			LTD 10	0.97	5	0.12	
	Bdnf Promoter II	Sc	Control	1.00	5	0.00	5.77E-01
			LTD 10	0.89	5	0.12	
		Sh	Control	1.00	5	0.00	5.77E-01
			LTD 10	0.95	5	0.10	
	Bdnf Promoter IV	Sc	Control	1.00	5	0.00	5.35E-03
			LTD 10	0.59	5	0.08	
		Sh	Control	1.00	5	0.00	1.89E-01
			LTD 10	0.87	5	0.10	
	Bdnf Promoter VI	Sc	Control	1.00	5	0.00	5.35E-03
			LTD 10	0.53	5	0.07	
		Sh	Control	1.00	5	0.00	5.77E-01
			LTD 10	0.99	5	0.07	

		Lentivirus		Mean	N	SEM
IgG	*Bdnf* Promoter I	Sc	Control	0.03	5	0.01
			LTD 10	0.02	5	0.01
		Sh	Control	0.03	5	0.01
			LTD 10	0.01	5	0.00
	Bdnf Promoter II	Sc	Control	0.01	5	0.00
			LTD 10	0.01	5	0.01
		Sh	Control	0.03	5	0.02
			LTD 10	0.02	5	0.01
	Bdnf Promoter IV	Sc	Control	0.01	5	0.00
			LTD 10	0.01	5	0.01
		Sh	Control	0.01	5	0.01
			LTD 10	0.06	5	0.03
	Bdnf Promoter VI	Sc	Control	0.04	5	0.03
			LTD 10	0.00	5	0.00
		Sh	Control	0.06	5	0.02
			LTD 10	0.03	5	0.01

Supplementary Table 8: Data and statistics corresponding to Supplementary Figure 4

B)

			AP	Mean	N	SEM
H3K27Me3S28p	*Bdnf* Promoter I	Control	-	0.23	4	0.01
			+	0.08	4	0.02
		LTD 10	-	0.26	4	0.03
			+	0.07	4	0.03
	Bdnf Promoter II	Control	-	0.22	4	0.02
			+	0.07	4	0.01
		LTD 10	-	0.35	4	0.05
			+	0.06	4	0.03
	Bdnf Promoter IV	Control	-	0.14	4	0.01
			+	0.04	4	0.01
		LTD 10	-	0.18	4	0.04
			+	0.04	4	0.02
	Bdnf Promoter VI	Control	-	0.21	4	0.02
			+	0.06	4	0.02
		LTD 10	-	0.28	4	0.01
			+	0.05	4	0.02

			AP	Mean	N	SEM
IgG	*Bdnf* Promoter I	Control	-	0.070	4	0.006
			+	0.049	4	0.022
		LTD 10	-	0.042	4	0.008
			+	0.079	4	0.018
	Bdnf Promoter II	Control	-	0.076	4	0.011
			+	0.051	4	0.018
		LTD 10	-	0.053	4	0.014
			+	0.069	4	0.019
	Bdnf Promoter IV	Control	-	0.067	4	0.007
			+	0.034	4	0.007
		LTD 10	-	0.064	4	0.013
			+	0.047	4	0.017
	Bdnf Promoter VI	Control	-	0.078	4	0.017
			+	0.050	4	0.021
		LTD 10	-	0.071	4	0.006
			+	0.052	4	0.003

C)

			AP	Mean	N	SEM	Mann-Whitney U Test
H3K27Me3	*Bdnf* Promoter I	Control	+	1.00	4	0.00	2.19E-01
		LTD 10		0.90	4	0.09	
	Bdnf Promoter II	Control	+	1.00	4	0.00	2.19E-01
		LTD 10		0.83	4	0.17	
	Bdnf Promoter IV	Control	+	1.00	4	0.00	1.39E-02
		LTD 10		0.66	4	0.07	
	Bdnf Promoter VI	Control	+	1.00	4	0.00	1.39E-02
		LTD 10		0.50	4	0.08	

			AP	Mean	N	SEM
IgG	*Bdnf* Promoter I	Control	+	0.006	4	0.002
		LTD 10		0.011	4	0.004
	Bdnf Promoter II	Control	+	0.006	4	0.002
		LTD 10		0.008	4	0.003
	Bdnf Promoter IV	Control	+	0.005	4	0.001
		LTD 10		0.014	4	0.007
	Bdnf Promoter VI	Control	+	0.004	4	0.001
		LTD 10		0.005	4	0.000

Supplementary Table 9: Data and statistics corresponding to Supplementary Figure 5

B)

	JMJD3 (pmols)	Mean	N	SEM	Mann-Whitney U Test
% of demethylated H3K27Me3S28p peptide	0	0.00	4	0.00	
	3.5	23.44	4	2.39	
	7	32.28	4	0.95	
	14	45.31	4	4.76	1.00E+00
% of demethylated H3K27Me3 peptide	14	39.44	4	6.24	

Supplementary Table 10: Data and statistics corresponding to Supplementary Figure 6

A)

Bonferroni adjustment	95% confidence interval	99% confidence interval	99.9% confidence interval
	(*) p < 1.67E-02	(**) p < 3.33E-03	(***) p < 3.33E-04

		Mean	N	SEM	Kruskal-Wallis Test	Mann-Whitney U Test		
						Control / LTD 10	Control / LTD 30	LTD10 / LTD 30
pS133-CREB	CNT	1.00	4	0.00	1.06E-02	1.39E-02	1.39E-02	8.33E-02
	LTD 10	1.88	4	0.28				
	LTD 30	1.35	4	0.09				

B)

Bonferroni adjustment	95% confidence interval	99% confidence interval	99,9% confidence interval
	(*) p < 1.25E-02	(**) p < 2.50E-03	(***) p < 2.50E-04

		KN93	Mean	N	SEM	Kruskal-Wallis Test	Mann-Whitney U Test					
							Control/LTD10	Control/Control +	Control/LTD10+	LTD10/Control+	LTD10/LTD10+	Control+/LTD10+
pS133-CREB	CNT	-	1.00	7	0.00	7.32E-03	8.29E-04	6.33E-01	1.52E-01	1.81E-02	2.53E-02	4.06E-01
	LTD10	-	1.79	7	0.16							
	CNT	+	1.09	7	0.14							
	LTD10	+	1.29	7	0.17							

C)

Bonferroni adjustment	95% confidence interval	99% confidence interval	99,9% confidence interval
	(*) p < 1.25E-02	(**) p < 2.50E-03	(***) p < 2.50E-04

		Cheleritryne	Mean	N	SEM	Kruskal-Wallis Test	Mann-Whitney U Test					
							Control/LTD10	Control/Control +	Control/LTD10+	LTD10/Control+	LTD10/LTD10+	Control+/LTD10+
pS133-CREB	CNT	-	1.00	7	0.00	3.41E-03	8.29E-04	6.33E-01	6.33E-01	2.68E-03	8.81E-03	8.48E-01
	LTD10	-	1.79	7	0.16							
	CNT	+	1.08	7	0.13							
	LTD10	+	1.05	7	0.16							

D)

Bonferroni adjustment	95% confidence interval	99% confidence interval	99,9% confidence interval
	(*) p < 1.25E-02	(**) p < 2.50E-03	(***) p < 2.50E-04

		H89	Mean	N	SEM	Kruskal-Wallis Test	Mann-Whitney U Test					
							Control/LTD10	Control/Control +	Control/LTD10+	LTD10/Control+	LTD10/LTD10+	Control+/LTD10+
pS133-CREB	CNT	-	1.00	7	0.00	3.31E-03	8.29E-04	6.33E-01	6.33E-01	8.81E-03	2.68E-03	7.49E-01
	LTD10	-	1.79	7	0.16							
	CNT	+	1.02	7	0.17							
	LTD10	+	0.93	7	0.17							

E)

Bonferroni adjustment	95% confidence interval	99% confidence interval	99.9% confidence interval
	(*) p < 1.67E-02	(**) p < 3.33E-03	(***) p < 3.33E-04

		Mean	N	SEM	Kruskal-Wallis Test	Mann-Whitney U Test		
						Control / LTD 10	Control / LTD 30	LTD10 / LTD 30
H3K27Ac	CNT	1.00	6	0.00	5.89E-04	2.82E-03	2.09E-03	6.17E-03
	LTD 10	1.33	6	0.11				
	LTD 30	0.60	6	0.06				

F)

Bonferroni adjustment	95% confidence interval	99% confidence interval	99.9% confidence interval
	(*) p < 1.67E-02	(**) p < 3.33E-03	(***) p < 3.33E-04

		Mean	N	SEM	Kruskal-Wallis Test	Mann-Whitney U Test		
						Control / LTD 10	Control / LTD 30	LTD10 / LTD 30
H3K9Ac	CNT	1.00	6	0.00	7.25E-01	5.77E-01	5.77E-01	4.65E-01
	LTD 10	1.06	6	0.17				
	LTD 30	0.88	6	0.20				

G)

Bonferroni adjustment	95% confidence interval	99% confidence interval	99.9% confidence interval
	(*) p < 1.67E-02	(**) p < 3.33E-03	(***) p < 3.33E-04

		Mean	N	SEM	Kruskal-Wallis Test	Mann-Whitney U Test		
						Control / LTD 10	Control / LTD 30	LTD10 / LTD 30
H3K14Ac	CNT	1.00	6	0.00	1.63E-01	5.77E-01	9.47E-02	1.17E-01
	LTD 10	1.15	6	0.17				
	LTD 30	0.74	6	0.24				

H)

Bonferroni adjustment	95% confidence interval	99% confidence interval	99.9% confidence interval
	(*) p < 1.67E-02	(**) p < 3.33E-03	(***) p < 3.33E-04

		Mean	N	SEM	Kruskal-Wallis Test	Mann-Whitney U Test		
						Control / LTD 10	Control / LTD 30	LTD10 / LTD 30
H3K18Ac	CNT	1.00	6	0.00	1.88E-01	1.39E-02	1.00E+00	5.64E-01
	LTD 10	0.82	6	0.07				
	LTD 30	0.87	6	0.16				

Supplementary Table 11: Data and statistics corresponding to Supplementary Figure 7

	Mean	N	SEM	Mann-Whitney U Test
pS276-p65	1.00	6	0.00	3.05E-01
	1.10	6	0.11	

Supplementary Table 12: Data and statistics corresponding to Supplementary Figure 9

A)

		Mean	N	SEM
CDYL	*Bdnf* Promoter I	0.05	4	0.005
	Bdnf Promoter II	0.10	4	0.005
	Bdnf Promoter IV	0.03	4	0.006
	Bdnf Promoter VI	0.03	4	0.005

		Mean	N	SEM
IgG	*Bdnf* Promoter I	0.030	4	0.008
	Bdnf Promoter II	0.013	4	0.003
	Bdnf Promoter IV	0.019	4	0.003
	Bdnf Promoter VI	0.017	4	0.005

B)

Bonferroni adjustment	95% confidence interval	99% confidence interval	99.9% confidence interval
	(*) p < 1.67E-02	(**) p < 3.33E-03	(***) p < 3.33E-04

			Mean	N	SEM	Kruskal-Wallis Test	Mann-Whitney U Test		
							Control / LTD 10	Control / LTD 30	LTD10 / LTD 30
CDYL	*Bdnf* Promoter II	CNT	1.00	4	0.00				
		LTD 10	0.46	4	0.12	2.18E-02	1.39E-02	1.39E-02	1.00E+00
		LTD 30	0.48	4	0.01				

			Mean	N	SEM
IgG	*Bdnf* Promoter II	CNT	0.14	4	0.03
		LTD 10	0.23	4	0.07
		LTD 30	0.20	4	0.08

C)

Bonferroni adjustment	95% confidence interval	99% confidence interval	99.9% confidence interval
	(*) p < 1.67E-02	(**) p < 3.33E-03	(***) p < 3.33E-04

		Mean	N	SEM	Kruskal-Wallis Test	Mann-Whitney U Test		
						Control / LTD 10	Control / LTD 30	LTD10 / LTD 30
CDYL	CNT	1.00	5	0.00				
	LTD 10	0.73	5	0.09	3.66E-03	5.35E-03	5.35E-03	7.58E-02
	LTD 30	0.50	5	0.09				

Supplementary Table 13: Primer list

Rat

EXPRESSION PRIMERS

		Forward			Reverse	
Bdnf Exon I	5'-	TTTCAACATCGATGCCAGT	-3'	5'-	ATCCACCTTGGCGATTACAG	-3'
Bdnf Exon II	5'-	AGTCCATTCAGCACCTTGGA	-3'	5'-	CTACCACCTCGGACAAATCC	-3'
Bdnf Exon IV	5'-	AAATGGAGCTTCTCACTGAAGG	-3'	5'-	ATTGCATGGCGGAGGTAATA	-3'
Bdnf Exon VI	5'-	CAACAATGTGACTCCACTGC	-3'	5'-	CAACAATGTGACTCCACTGC	-3'
Jmjd3	5'-	CTGAAACTGCCTGCCTTCAT	-3'	5'-	CTAAGCATGTTGCCTGTGGA	-3'
Gapdh	5'-	ATGACTCTACCCACGGCAAG	-3'	5'-	GATCTCGCTCCTGGAAGATG	-3'
GusB	5'-	GCCAATGAGCCTGTCTCTTC	-3'	5'-	TCCAGTTCTTGGGGAATCTG	-3'
Pgk1	5'-	AATGATGCTTTTGGGACTGC	-3'	5'-	TCAAAAATCCACCAGCCTTC	-3'

CHIP PRIMERS

		Forward			Reverse	
Bdnf Promoter I	5'-	GCCTCTCGCCTAGTCATCAG	-3'	5'-	CCCCACAACTTTCCCTTTTC	-3'
Bdnf Promoter II	5'-	GGACTGGAGGGGGTTGTTAT	-3'	5'-	TCAGACAAGCATCAGCTTTGA	-3'
Bdnf Promoter IV	5'-	GTGAGTTCGCTAGGACTGGAA	-3'	5'-	GGCATTGCATGCTTTGTAGA	-3'
Bdnf Promoter VI	5'-	CTCCCACAGAACTTGGGTGT	-3'	5'-	TTTGCAACTCTCCCCATCTT	-3'
βActin	5'-	CATCGCCAAACTCTTCATCC	-3'	5'-	GAGCGAGAGAGAAAGCGAGA	-3'
hoxA1	5'-	TCTTGCGCACTGTACATTCA	-3'	5'-	CCTCCATAGGACCAGAGAAGAA	-3'

Mouse

EXPRESSION PRIMERS

		Forward			Reverse	
Bdnf Transcript I	5'-	TTTCAACATCGATGCCAGTT	-3'	5'-	ATCCACCTTGGCGACTACAG	-3'
Bdnf Transcript II	5'-	CCATCCACACGTGACAAAAC	-3'	5'-	TGCTCTAGACGGTTTCTTCCA	-3'
Bdnf Transcript IV	5'-	AAATGGAGCTTCTCGCTGAA	-3'	5'-	ATTGCATGGCGGAGGTAATA	-3'
Bdnf Transcript VI	5'-	GAGACCCGGTTCCTTCAACT	-3'	5'-	CTTTCTCGTCTGCCCCAAG	-3'
Gapdh	5'-	CTCCCACTCTTCCACCTTCG	-3'	5'-	CATACCAGGAAATGAGCTTGACAA	-3'
GusB	5'-	AGCCGCTACGGGCGTCG	-3'	5'-	GCTGCTTCTTGGGTGATGTCA	-3'
Pgk1	5'-	TACCTGCTGGCTGGATGG	-3'	5'-	CACAGCCTCGGCATATTTCT	-3'

CHIP PRIMERS

		Forward			Reverse	
Bdnf Promoter I	5'-	GCCTCTCGCCTAGTCACAG	-3'	5'-	CCCCACAACTTTCCCTTTTC	-3'
Bdnf Promoter II	5'-	CTGGCTGTTCAAAGCTGATG	-3'	5'-	GGATGGAATACAAGACGGTTG	-3'
Bdnf Promoter IV	5'-	AAAAACGGTCCAAAGACCAC	-3'	5'-	TCACTAAGCCCCCTTCCTCT	-3'
Bdnf Promoter VI	5'-	AAACCAGGGGAGAAAGATTTG	-3'	5'-	GGAGGAAGCGAGTGTGAGTC	-3'
βActin	5'-	CCTGTACATCTGGGCCTACG	-3'	5'-	ATGAAGAGTTTTGGCGATGG	-3'
hoxA1	5'-	GCCACAAGAGAGCCAGGA	-3'	5'-	TGAACTGGCAAGAGGTGAGA	-3'
Cox1	5'-	GAGAGGGGGAAAAGTTGGTG	-3'	5'-	TGTCTTTCCGCTTAGGCTTT	-3'

Supplementary Table 14: Antibodies list

Antibody against	Raised in (Isotype)	ChIP	WB	IP	DB	Origin & Ref.
Hitone H3	Rabbit (IgG)	0.1 µg	1:1000	-	-	Cell Signaling ref. 4499
Trimethyl-Histone H3 (Lys4)	Rabbit (Serum)	4 µL	-	-	-	Active Motif ref. 39159
Acetyl-Histone H3 (Lys9)	Rabbit (IgG)	-	1:1000	-	-	Cell Signaling ref. 9649
Trimethyl-Histone H3 (Lys9)	Rabbit (IgG)	4 µg	-	-	-	Abcam ref. ab8898
Acetyl-Histone H3 (Lys14)	Rabbit (IgG)	-	1:1000	-	-	Cell Signaling ref. 4318
Acetyl-Histone H3 (Lys18)	Rabbit (IgG)	-	1:1000	-	-	Cell Signaling ref. 9675
Acetyl-Histone H3 (Lys27)	Rabbit (IgG)	0.1 µg	1:1000	-	-	Cell Signaling ref. 4353
Trimethyl-Histone H3 (Lys27)	Rabbit (IgG)	4 µg	-	-	1:1000	Millipore ref. 07-449
Trimethyl-Phsopho-Histone H3 (Lys27Ser28)	Rabbit (Serum)	5 µL	-	-	1:1000	Diagenode ref. CS-091-100 (Lot 1)
JMJD3	Rabbit (IgG)	10 µg	1:250	-	-	Abcam ref. ab38113
EZH2	Rabbit (IgG)	10 µg	-	-	-	Abcam ref. ab3748
Phospho-CREB (Ser133)	Rabbit (IgG)	-	1:1000	-	-	Cell Signaling ref. 9191
CREB	Rabbit (IgG)	10 µg	1:1000	-	-	Abcam ref. ab31387
CBP	Rabbit (IgG)	5 µg	-	-	-	Santa Cruz ref. sc-583
CBP	Mouse (IgG)	-	1:500	-	-	Abcam ref. ab79270
p-38	Rabbit (IgG)	2 µg	1:1000	-	-	Abcam ref. ab170099
Phospho-p38 (Thr180/Tyr182)	Rabbit (IgG)	-	1:1000	-	-	Cell Signaling ref.4511
CDYL	Rabbit (IgG)	5 µg	1:500	-	-	Abcam ref. ab5188
FLAG-M2	Mouse (IgG1)	-	-	25µL	-	Sigma ref. F3165
normal rabbit IgG	Rabbit (IgG)		assay dependet			Santa Cruz ref. sc-2027
normal mouse IgG	Mouse (IgG)		assay dependet			Santa Cruz ref. sc-2025

Supplementary Methods

Cell Cultures

HEK293T cells (ATCC) and Mouse Embrionic Fibroblasts (MEFs) WT and p38 KO[1] (kindly provided by Dr. A. R. Nebreda; Institute for Research in Biomedicine (IRB Barcelona), Barcelona, Spain) were grown with Dulbecco's modified Eagle's medium (DMEM) supplemented with 10% FBS and antibiotics (100 units/mL penicillin and 100 mg/mL streptomycin). DMEM was supplemented with 2 mM L-glutamine for MEFs culture. Cells were grown at 37ºC in a humidified atmosphere of 5% CO_2.

Drug treatments

Stock solutions of Forskolin (50 mM, Tocris Bioscience ref. 1099) and Rolipram (100µM, Tocris Bioscience ref. 0905) were prepared in dimethyl sulfoxide (Sigma). Stock solutions of CaMKII inhibitor (KN93 phosphate; 20 mM; Tocris ref. 5215), Protein Kinase A (PKA) inhibitor (H89 dihydrochloride; 25 mM; Tocris ref. 2910) and Portein Kinase C (PKC) inhibitor (Chelerytrine chloride; 10 mM; Tocris ref. 1330) were prepared in Milli-Q water. Hippocampal neurons were treated with 30 µM KN93 (20 min), 50 µM H89 (20 min) or 10 µM Chelerytrine (20 min) prior to NMDA-LTD induction. LTD was induced by 20 µM NMDA (Sigma-Aldrich) for 5 minutes, and the medium was replaced. Samples were collected 5 or 25 minutes after LTD induction. Samples without LTD induction were used as a control. HEK293T cells were treated with 50 µM Forskolin and 0.1 µM Rolipram for 15 min.

p38 antibody validation for ChIP

Wild type and p38 KO MEFs were traded with 100 mM NaCl for 45 minutes in order to induce hyperosmotic stress, a treatment known to activate p38 and promote its recruitment to different genes promoters[2]. In Supplementary Figure 2B we show p38 is recruited to *Cox2* promoter upon hyperosmotic stimulation in WT MEFs and that this recruitment does not take place in p38 KO MEFs. These results suggest that the antibody used is ChIP grade for p38.

H3K27Me3S28p antibody crossreactivity

Inreasing amounts of Histone H3 peptides containing different marks (Diagenode: H3K9Me3S28p ref. C16000128, H3K27 unmodified ref.C16000998, H3K27Me3 ref. C16000069, H3K27Me3S28p ref. C16000091) mixtures were loaded onto a nitrocellulose membrane using a dot-blot (Bio-Rad, ref. 1706545). Membrane were blocked 1h with 5% BSA in TTBS and incubated O/N with H3K27Me3S28p antibody (Supplementary Table 10). Peroxidase-conjugated polyclonal goat anti-rabbit (Dako ref. P0448) was used as secondary antibody at 1:5000 for 1 h at RT in TTBS containing 5% BSA. Dots were visualized with Super Signal (Thermoscientific) in an ImageQuant LAS 4000 Mini (GE Healtcare Life Sciences).

Histone H3 peptides dephosphorylation

20 μL of Histone H3 peptides H3K27Me3 and H3K27Me3S28p [10 μM] (Diagenode, H3K27Me3 ref. C16000069, H3K27Me3S28p ref. C16000091) were incubated for 1hour at 37ºC with 4 units of Alkaline Phosphatase (CIP; New England BioLabs ref. M0290S) and in a buffer containing 50 mM HEPES (pH 7.9) and MgCl 10 mM. 20 μL of Histone H3 peptides H3K27Me3 and H3K27Me3S28p [10 μM] control non dephosphorylated peptides were incubated in a buffer containing 50 mM HEPES (pH 7.9) and MgCl 10 mM plus phosphatase inhibitors. After treatment mixtures were loaded onto a nitrocellulose membrane using a dot-blot (Bio-Rad, ref. 1706545). Membrane were blocked 1h with 5% BSA in TTBS and incubated O/N with H3K27Me or H3K27Me3S28p antibodies (Supplementary Table 10). Peroxidase-conjugated polyclonal goat anti-rabbit (Dako ref. P0448) was used as secondary antibody at 1:5000 for 1 h at RT in TTBS containing 5% BSA. Dots were visualized with Super Signal (Thermoscientific) in an ImageQuant LAS 4000 Mini (GE Healtcare Life Sciences).

Chromatin dephosphorylation

Samples were collected and sonicated in Sof Lysis Buffer and SDS Lysis buffer with protease inhibitors and without EDTA, respectively (see ChIP protocol in Methods section). Finally chromatin was adjusted at 1μg/μL with SDS Lysis Buffer without EDTA. Samples were incubated for 1hour at 37ºC with 4 units of Alkaline Phosphatase *per* μg of chromatin (CIP; New England BioLabs ref. M0290S) and MgCl 10 mM. After 1 hour phosphatase inhibitor was added. Control non-de-phosphorylated samples were incubated 1 hour at 37ºC with MgCl 10 mM and phosphatase inhibitors. After de-phosphorylation treatment we followed ChIP protocol by diluting samples 1/10 with Dilution Buffer (see ChIP protocol as it follows in Methods section).

Lentiviral production and neuronal infection

Packaging kit and ShRNA plamid against JMJD3 or srcamble were purchased from Origene (ShRNAs Ref. TL707358; ShRNA lentiviral packaging kit Ref. TR30022). Lentiviral particles were produced in HEK293T according to Origene instructions. Neurons were infected at 11 DIV and medium was fully changed with neuronal conditioned medium at 12 DIV. Experiments were performed at 15 DIV.

JMJD3 antobody validation for ChIP

In the Supplementary Figure 4A-C we show the experiments designed to assess the specificity of the antibody used. In the panel A we show that treatment of hippocampal neurons in culture with a lentivirus expressing a shRNA to knock down JMJD3 leads to a 50% reduction in the amount of the specific mRNA compared to cultures infected with leniviral particles expressing a scrambled shRNA. In the panel B, the Western blot show that JMJD3 knock down resulted in a 75% reduction of JMJD3 compared to scrambled-shRNA treated controls. The ChIP experiments performed with the Abcam, ab38113 antibody show that the enrichment of JMJD3 at the *Bdnf* promoters is lost in cultures where JMJD3 was knocked down. These results suggest that the antibody used is ChIP grade for JMJD3.

In vitro JMJD3 demethylase activity assay:

Histone demethylase assay was performed as described by Hong et al. (2007)[3] with minor modifications. Briefly, 10 μM of H3K27Me3 (Diagenode) peptide was incubated with 0 or 14 pmol of the active human recombinant JMJD3 (Sigma-Aldrich, ref. SRP0162) in 20μ of demethylase assay buffer [50 mM HEPES (pH 7.9), 50 mM KCl, 50 mM $(NH_4)_2Fe(SO_4)_2 \cdot 6(H_2O)$, 1 mM α-ketoglutarate, and 2 mM ascorbic acid]. 10 μM H3K27Me3S28p peptide (Diganeode) was incubated with increasing concentrations (0, 3.5, 7 and 14 pmol) of the active human recombinant JMJD3 in 20μ of the demethylase assay buffer. Demethylation assay mixtures were kept for 5 h at 37°C with soft agitation (400 rpms). Assay mixtures were loaded onto a nitrocellulose membrane using a dot-blot (Bio-Rad, ref. 1706545). Membrane were blocked 1h with 5% BSA in TTBS and incubated O/N with specific antibodies (Supplementary Table 10). Peroxidase-conjugated polyclonal goat anti-rabbit (Dako ref. P0448) was used as secondary antibody at 1:5000 for 1 h at RT in TTBS containing 5% BSA. Dots were visualized with Super Signal (Thermoscientific) in an ImageQuant LAS 4000 Mini (GE Healtcare Life Sciences). Dots were quantified with ImageJ.

Protein identification by Western Blot

Neurons were lysed on ice with a solution containing 1 M Tris-HCl, 1% Nonidet P-40, 150 mM NaCl, 5 mM EDTA, 1 mM sodium orthovanadate, 1 mM dithiotreitol, pH 7.4, protease inhibitor cocktail (Roche) and phosphatase inhibitor cocktail 2 (Sigma-Aldrich). Protein concentration was determined by Pierce® BCA protein assay kit (Thermoscientific). Protein samples were electrophoretically resolved within 10% Tris-HCl gels, 15% Tris-HCl gels for Histones, and afterwards transferred to nitrocellulose membranes using iBlot Gel Transfer System (Invitrogen). Membranes were blocked in Tween 20-Tris buffer solution (TTBS: 0.1% v/v Tween 20, 100 mM Tris-HCl, 150 mM NaCl, pH 7.5), containing 5% BSA (Sigma-Aldrich), and incubated overnight at 4°C with the antibodies (Supplementary Table 10), diluted in 5% BSA in TTBS. Peroxidase-conjugated polyclonal goat anti-rabbit (Dako ref. P0448) or peroxidase-conjugated polyclonal rabbit anti-mouse (Dako ref. P0260) were used as secondary antibodies at 1:5000 for 1 h at RT in TTBS containing 5% BSA. Bands were visualized with Super Signal (Thermoscientific) in an ImageQuant LAS 4000 Mini (GE Healtcare Life Sciences). Bands were quantified with ImageJ.

Immunoprecipitation

HEK293T cells were transfected with Lipofectamine 2000 according to the manufacturer instructions (Life technologies, ref. 11668) using a plasmid that drive the expression of 6xMyc-JMJD3-FLAG (Addgene, ref. 17440)[3] and a plasmid that drive the expression of CBP when co-transfectred with a Tet-Off containing plasmid[4] (kindly provided by Dr A. Barco; Instituto de Neurociencias, Universidad Miguel Hernández-Consejo Superior de Investigaciones Científicas, Alicante, Spain). HEK293T were treated with Forskolin (50μM) and Rolipram (0.1 μM) for 15 min and crosslinked with dithiobis(succinimidyl-propionate) (DSP) 5 mM (Life technologies ref. 22585) for 30 min at RT. Reaction was stopped by adding Tris ph7.5 at final concentration 20 mM for 15 min. Cells were washed two times with cold PBS and homogenized in STEN Buffer plus inhibitors and sonicated until clarification. Protein concentration was determined by Pierce® BCA protein assay kit (Thermoscientific). Samples were pre-cleared with 40

μL protein A/G agarose beads and 10 μg of normal IgG for each 200 μg of protein. Samples were placed in a rotor for 1–3 h at 4ºC. The mixture was centrifuged and the supernatant was collected. 200 μg of protein were used for each IP assay. FLAG antibody (Supplementary Table 10) was added to the lysate, incubated on a rotor O/N at 4ºC. Immune complexes were precipitated by the addition protein A/G agarose beads. As a negative control, non-immune mouse IgG was used in place of specific antibodies. Immuneprecipitated complexes were washed five times with STEN Buffer and one time with PBS. Complexes were eluted with Laemmli Buffer 2X (120 mM Tris pH 6.8, 4% SDS, 20% glycerol, 2% ß-mercaptoethanol and 0,02% bromophenol blue) for 10 min at 95ºC. Proteins were identified by Western Blot (resolved within 5% Tris-HCl gels).

Statistical analysis

All values are presented as mean ± SEM. Mann-Whitney U test and Kruskal-Wallis followed by Mann-Whitney U test multiple comparisons with Bonferroni adjustment were used for statistical analysis of the data using SPSS6 (SPSS6 Statistics, IBM.). P values lower than 0.05 were considered significant. In the figures asterisks indicate P values as follows: *< 0.05; **< 0.01; ***< 0.001.

Supplementary references

1. Porras, A. *et al*. P38 alpha mitogen-activated protein kinase sensitizes cells to apoptosis induced by different stimuli. *Mol. Biol. Cell* **15,** 922–33 (2004).

2. Ferreiro, I. *et al*. Whole genome analysis of p38 SAPK-mediated gene expression upon stress. *BMC Genomics* **11,** 144 (2010).

3. Hong, S. *et al*. Identification of JmjC domain-containing UTX and JMJD3 as histone H3 lysine 27 demethylases. *Proc. Natl. Acad. Sci. U. S. A.* **104,** 18439–44 (2007).

4. Valor, L. M. *et al*. Ablation of CBP in forebrain principal neurons causes modest memory and transcriptional defects and a dramatic reduction of histone acetylation but does not affect cell viability. *J. Neurosci.* **31,** 1652–63 (2011).
